# Effect of Avoiding Cow's Milk Formula at Birth on Prevention of Asthma or Recurrent Wheeze Among Young Children: Extended Follow-up From the ABC Randomized Clinical Trial

**DOI:** 10.1001/jamanetworkopen.2020.18534

**Published:** 2020-10-02

**Authors:** Hiroshi Tachimoto, Eiji Imanari, Hidetoshi Mezawa, Mai Okuyama, Takashi Urashima, Daishi Hirano, Noriko Gocho, Mitsuyoshi Urashima

**Affiliations:** 1 Division of Molecular Epidemiology, Jikei University School of Medicine, Tokyo, Japan; 2 Department of Pediatrics, Jikei University School of Medicine, Tokyo, Japan

## Abstract

**Importance:**

Children with food allergies may develop asthma or recurrent wheeze.

**Objective:**

To evaluate whether asthma or recurrent wheeze among children were changed by avoiding supplementing breastfeeding (BF) with cow’s milk formula (CMF) in the first 3 days of life.

**Design, Setting, and Participants:**

This randomized, unmasked, clinical trial was conducted at 1 university hospital in Japan beginning October 2013 with follow-up examinations occurring until January 2020. A total of 312 newborns at risk for atopy were randomized and assigned to either BF with or without amino acid–based elemental formula (EF) or BF with CMF, with follow-up examinations for participants showing signs of atopy conducted at 24 months. Follow-up examinations ran through January 2020.

**Interventions:**

Immediately after birth, newborns were randomly assigned (1:1 ratio) to either breastfeeding with or without amino acid–based elemental formula for at least the first 3 days of life (no CMF group) or breastfeeding supplemented with CMF (≥5 mL/d) from the first day of life to 5 months of age (CMF group).

**Main Outcomes and Measures:**

Asthma or recurrent wheeze diagnosed by the pediatric allergy specialists of this trial; subgroups were stratified by serum levels of 25-hydroxyvitamin D and IgE.

**Results:**

Of 312 infants (156 [50.0%] randomized to the no CMF group), 302 (96.8%) were followed up at their second birthday: 77 of 151 (51.0%) in the no CMF group and 81 of 151 (53.6%) in the CMF group underwent extended follow-up because of having atopic conditions. Asthma or recurrent wheeze developed in 15 (9.9%) of the children in the no CMF group, significantly less than the children in the CMF group (27 [17.9%]; risk difference, −0.079; 95% CI, −0.157 to −0.002). In participants with vitamin D levels above the median at 5 months of age, asthma or recurrent wheeze developled in 5 (6.4%) children in the no CMF group, significantly less than in the children in the CMF group (17 [24.6%]; risk difference, −0.182; 95% CI, −0.298 to −0.067; *P *for interaction = .04). In the highest quartile group of total IgE at age 24 months, asthma or recurrent wheeze developed in 2 children (5.3%) in the no CMF group, significantly less than the children in the CMF group (14 [43.8%]; risk difference, −0.385; 95% CI, −0.571 to −0.199; *P* for interaction = .004).

**Conclusions and Relevance:**

The findings of this study suggest that avoiding CMF supplementation in the first 3 days of life has the potential to reduce the risk of asthma or recurrent wheeze in young children, especially among those with high vitamin D or high IgE levels.

**Trial Registration:**

umin.ac.jp/ctr Identifier: UMIN000011577

## Key Points

**Question:**

Is the risk of asthma or recurrent wheeze in young children decreased by avoiding supplementation with small amounts of cow’s milk formula at birth?

**Findings:**

This randomized clinical trial of 312 children at risk for atopy examined risks of food allergy by the second birthday in extended follow-up examinations. Asthma or recurrent wheeze developed in significantly fewer children breastfed with or without amino acid–based elemental formula for at least the first 3 days of life than in children breastfed with supplementation from cow’s milk formula (≥5 mL/d) from the first day.

**Meaning:**

The findings of this study suggest that asthma or recurrent wheeze can be prevented by avoiding cow’s milk formula supplementation at birth.

## Teaser

This follow-up of the ABC randomized clinical trial compares the risk of asthma or recurrent wheeze among young children who were randomized as infants to either breastfeeding with cow’s milk formula supplementation or breastfeeding with or without elemental formula supplementation in the first few days of life.

## Introduction

Asthma is among the most common chronic diseases in the world, and its prevalence has been increasing during the past 60 years.^1^ Asthma with onset in early adulthood may have its origins in early childhood.^2,3^ Therefore, the primary prevention of childhood asthma may be key in mitigating the burden of this disease. A 1989 study of patients with hay fever^4^ showed an increase in the prevalence of atopic disease can in part be explained by the hygiene hypothesis that smaller family size, improvements in household amenities, and higher standards of personal cleanliness in wealthier households have reduced the opportunity for cross-infection in young families. Gupta et al^5^ reported that eczema and having a lower number of siblings were associated with increased risks of childhood food allergy and asthma. However, this study reported that food allergy was associated with skin infection, whereas asthma was associated with respiratory syncytial virus infection. A 2016 meta-analysis^6^ demonstrated that food sensitization in the first 2 years of life was associated with an increased risk of asthma or recurrent wheeze. A 2018 study^7^ found that food allergy in infancy is also associated with an increased risk of asthma or recurrent wheeze in toddlers, irrespective of whether the early food allergy resolves.

The World Health Organization recommends breastfeeding (BF) for at least 6 months after birth because of general health benefits for the child. However, a 2010 cohort study^8^ demonstrated that the incidence of cow’s milk allergy was lower in infants who began receiving regular cow’s milk formula (CMF) within the first 14 days of life, and thus, the authors of this study recommended CMF supplementation at birth. Many Japanese maternity wards encourage BF, but some (including Jikei University Hospital, where this trial was performed) allow mothers or nurses to supplement BF with small amounts of CMF (eg, ≥5 mL) approximately 6 to 10 hours after birth (or earlier in some cases) depending on maternal preferences.

We previously conducted a randomized clinical trial (RCT) (the Atopy Induced by Breastfeeding or Cow’s Milk Formula [ABC] trial), in which newborns were assigned to 1 of the following groups immediately after birth: (1) BF with or without an amino acid–based elemental formula (EF), avoiding supplementation with CMF for at least the first 3 days of life (ie, the no CMF group), or (2) BF supplemented with a small amount of CMF (≥5 mL/d) from the first day of life (ie, the CMF group).^9^ Participants then underwent follow-up until their second birthday. Because asthma and recurrent wheeze tend to be diagnosed later than food allergy in infants, participants who needed medical care for atopic conditions (eg, eczema, atopic dermatitis, food allergy, recurrent wheeze, and/or atopic sensitization) underwent extended follow-up to a maximum age 6 years. Selection bias would be expected because asthma or recurrent wheeze was originally set as 1 of the prespecified exploratory outcomes and was planned to be reported separately. Therefore the aim was to examine whether asthma or recurrent wheeze is preventable by avoiding CMF for at least the first 3 days of life with extended follow-up of participants in the ABC trial.

## Methods

### Trial Design

Newborn infants at risk for atopy born in Jikei University Hospital, with at least 1 of the father, mother, or siblings having current and/or past atopic diseases (eg, asthma, atopic dermatitis, food allergy, allergic rhinitis, or hay fever), were randomly assigned immediately after birth to avoid supplementation with CMF for at least the first 3 days of life (the no CMF group), or to BF supplemented with CMF (≥5 mL/d) (the CMF group). Each group adhered to these conditions until age 5 months and then followed up to their second birthdays. There was no restrictions on the mother’s diet during the intervention to age 5 months. Although the ABC trial initially ended when the last participant reached her second birthday, participants who needed medical care for atopic conditions (eg, eczema, atopic dermatitis, food allergy, recurrent wheeze, and/or atopic sensitization) at their second birthday underwent extended follow-up to a maximum age of 6 years. Thus, a diagnosis of asthma or recurrent wheeze during preschool age was set as 1 of the prespecified exploratory outcomes in the protocol of the ABC trial. Serum levels of 25-hydroxyvitamin D (25[OH]D),^10^ total IgE, and antigen-specific IgE levels were measured at age 5 months and 24 months. The trial protocol was approved by the ethics committee of the Jikei University School of Medicine, and parents of infants provided written informed consent. The full protocol for this trial is available in Supplement 1. This study followed the Consolidated Standards of Reporting Trials (CONSORT) reporting guideline for RCTs.

### Outcome

The primary outcome of this study was the incidence of asthma or recurrent wheeze based on the asthma predictive index.^11^ This outcome had been prespecified in the original protocol as follows: (1) frequent wheezing, and (2) either 1 of 3 major risk factors (ie, parental history of asthma; eczema diagnosed by pediatricians involved in this trial and evaluated using the Severity Scoring of Atopic Dermatitis index^12^; or sensitization with elevated mite-specific IgE ≥0.10 U_A_/mL) or 2 of 3 minor risk factors (ie, eosinophilia [≥4%]; wheezing diagnosed when inhalation with a β-stimulant improved the clinical signs of wheeze and dyspnea effectively; or allergic rhinitis diagnosed, if signs and symptoms of rhinitis continued for 1 month and mite-specific IgE ≥0.10 U_A_/mL, by the pediatricians involved in this trial). The pediatrician who diagnosed asthma or recurrent wheeze was an allergy specialist and totally masked to the allocation group.

### Follow-up

**Figure 1.**
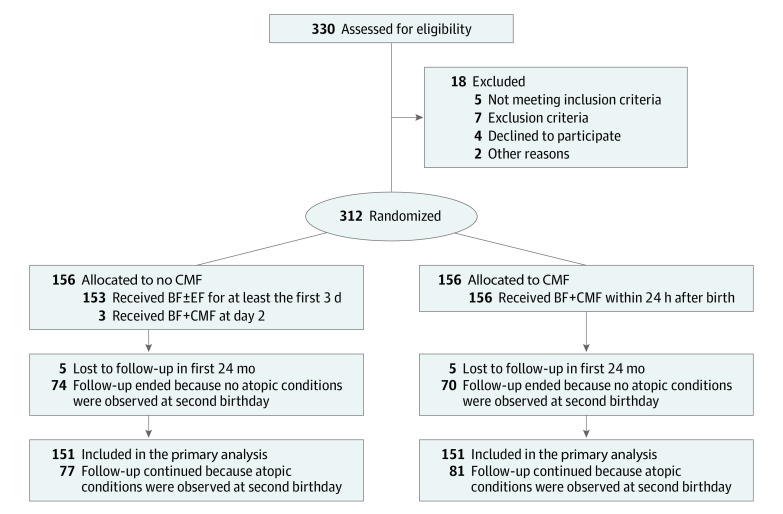
Patient Flow Through the Atopy Induced by Breastfeeding or Cow’s Milk Formula (ABC) Trial BF indicates breastfeeding; CMF, cow’s milk formula; EF, elemental formula.

Enrollment began in October 2013, and follow-up for all participants to assess the risk of food allergy initially ended on May 31, 2018. Follow-up for participants without atopic conditions was ended at their second birthday. However, participants who needed medical care for atopic conditions (eg, eczema, atopic dermatitis, food allergy, recurrent wheeze, and/or atopic sensitization) underwent extended follow-up at the outpatient clinic of Jikei University Hospital after their second birthday until January 4, 2020. After excluding 18 families, 312 of 330 infants (94.5%) were randomized to the no CMF group (156 infants) or the CMF group (156 infants) in the first 3 days of life (Figure 1). One hundred fifty-one children (96.8%) in the no CMF group and 151 children (96.8%) in the CMF group could be followed-up at their second birthday. In the no CMF group, 77 infants (51.0%) developed atopy and underwent extended follow-up due to having atopic conditions for a median of 3.0 years (interquartile range [IQR], 2.0-4.2 years; maximum, 5.6 years). In the CMF group, 81 infants (53.6%) developed atopy and underwent extended follow-up for a median of 3.3 years (IQR, 2.1-4.3 years; maximum, 6.0 years). These differences were not statistically significant.

### Statistical Analysis

All participants who underwent randomization and were followed-up to at least their second birthdays were included in this analysis. Asthma or recurrent wheeze was assessed according to the randomization group even in cases that did not adhere to the intervention as an intention-to-treat analysis. Skewness and kurtosis tests were used to assess the normality of the distributions. Parametric and nonparametric continuous variables with normal and nonnormal distributions were compared between 2 groups and among 4 groups by the *t* test and the Mann-Whitney test, and analysis of variance and the Kruskal-Wallis test, respectively. Dichotomous variables were compared between the groups by the χ^2^ test. Spearman rank correlation coefficient (ρ) with linear regression was used to quantify the strengths of associations between 2 continuous variables (strong, ρ ≥ 0.4; moderate, ρ < 0.4 and ≥0.2; and weak, ρ < 0.2). Effects of the intervention on the cumulative incidence of asthma or recurrent wheeze were estimated using risk differences (RDs) and risk ratios (RRs) with 95% CIs. When both sides of the 95% CI of RD were more or less than 0 or both sides of the 95% CI of RR did not include 1, the risk estimate was considered to be significant. Prespecified subgroups were stratified at the median and tertiles of 25(OH)D levels sampled at age 5 and 24 months (ie, 25[OH]D <29 ng/mL at 5 months, ≥29 ng/mL at 5 months, <21 ng/mL at 24 months, and ≥21 ng/mL at 24 months; to convert 25[OH]D to nanomoles per liter, multiply by 2.496). To clarify whether the intervention significantly affected these subgroups, *P* values for interaction were analyzed using the Mantel-Haenszel test. All reported *P* values were 2-sided and *P* < 0.05 was considered significant. These analyses were not corrected for multiple comparisons. All data were analyzed using Stata version 14.0 (StataCorp).

In post hoc analysis, subgroup analyses were performed. Infants were divided by quartile of serum levels of total IgE at age 24 months (ie, ≤5 IU/mL, 6-13 IU/mL, 14-49 IU/mL, and ≥50 IU/mL), by the median of total IgE levels at age 5 months (ie, <10 IU/mL and ≥10 IU/mL), and by positivity (≥0.1 U_A_/mL) for allergen-specific IgE at age 5 and 24 months.

Infants in the no CMF group were further divided into 3 subgroups: (1) remained on BF with or without EF at age 5 months; (2) switched from EF to CMF on day 15 or after; and (3) switched from EF to CMF on day 14 or before. The incidences of asthma or recurrent wheeze were then compared among these 3 groups.

## Results

### Effects of Avoiding CMF at Birth on Asthma or Recurrent Wheeze

The characteristics of the 302 participants who underwent randomization and were followed up to at least their second birthdays were compared among 4 groups: (1) no CMF without atopic conditions; (2) no CMF with atopic conditions; (3) CMF without atopic conditions; and (4) CMF with atopic conditions (Table). Participants’ characteristics were not different among these 4 groups, except that placental weight was heavier in infants in the no CMF group with atopic conditions than in the no CMF group without atopic conditions (mean [SD] weight, 615 [109] g vs 553 [86] g). Total IgE levels were not different between the no CMF (median [IQR], 13 [6-46] IU/mL) and the CMF (median [IQR], 15 [6-46] IU/mL; *P* = .45) groups nor between the no CMF with atopic conditions (median [IQR], 22 [6-69] IU/mL) and the CMF with atopic conditions (median [IQR], 30 [11-91] IU/mL; *P* = .11) groups, whereas they were significantly different in each group between participants with and without atopic conditions (no CMF group difference: median [IQR], 26 [9-79] IU/mL; CMF group difference: median, 7 [4-24.5] IU/mL; *P* < .001).

**Table.** Participants’ Characteristics

Perinatal variables before or at randomization	Participants, No. (%)			
	No CMF group (n = 151)		CMF group (n = 151)	
	Without atopy (n = 74)^a^	With atopy (n = 77)^b^	Without atopy (n = 70)^a^	With atopy (n = 81)^b^
Parental age, mean (SD), y				
Father	37.4 (5.4)	36.8 (6.8)	37.7 (6.9)	37.5 (6.8)
Mother	35.4 (4.2)	34.8 (4.9)	35.0 (4.5)	35.4 (4.2)
Atopic disease of mother				
Bronchial asthma				
Current	5 (6.8)	4 (5.2)	3 (4.3)	3 (3.7)
Previous	9 (12.2)	14 (18.2)	5 (7.1)	15 (18.5)
Atopic dermatitis				
Current	9 (12.2)	9 (11.7)	4 (5.7)	9 (11.1)
Previous	11 (14.9)	20 (26.0)	13 (18.6)	18 (22.2)
Food allergy				
Current	15 (20.3)	13 (16.9)	3 (4.3)	10 (12.4)
Previous	6 (8.1)	12 (15.6)	7 (10.0)	8 (9.9)
Allergic rhinitis				
Current	18 (24.3)	18 (23.4)	16 (22.9)	23 (28.4)
Previous	6 (8.1)	11 (14.3)	6 (8.6)	7 (8.6)
Pollen allergy				
Current	42 (56.8)	41 (53.3)	41 (58.6)	46 (56.8)
Previous	8 (10.8)	3 (3.9)	4 (5.7)	7 (8.6)
Atopic disease of father				
Bronchial asthma				
Current	3 (4.1)	0	6 (8.6)	4 (4.9)
Previous	10 (13.5)	12 (15.6)	10 (14.3)	13 (16.1)
Atopic dermatitis				
Current	1 (1.4)	6 (7.8)	5 (7.1)	8 (9.9)
Previous	5 (6.8)	9 (11.7)	10 (14.3)	17 (17.3)
Food allergy				
Current	12 (16.2)	8 (10.4)	7 (10.0)	10 (12.4)
Previous	3 (4.1)	1 (1.3)	5 (7.1)	4 (4.9)
Allergic rhinitis				
Current	19 (25.7)	18 (23.4)	19 (27.1)	19 (23.5)
Previous	7 (9.5)	4 (5.2)	8 (11.4)	9 (11.1)
Pollen allergy				
Current	40 (54.1)	36 (46.8)	37 (52.9)	39 (48.2)
Previous	4 (5.4)	5 (6.5)	1 (1.4)	2 (2.5)
Perinatal status				
Gestational time, median (IQR), wk	39 (38-39)	39 (38-39)	39 (38-39)	39 (38-39)
Cesarean delivery				
Planned	10 (13.5)	7 (9.1)	8 (11.4)	7 (8.6)
Emergency	9 (12.2)	11 (14.3)	9 (12.9)	12 (14.8)
Female sex	41 (55.4)	36 (46.8)	35 (50.0)	41 (50.6)
Apgar score at 5 min, points				
8	2 (2.9)	2 (2.7)	3 (4.4)	6 (7.5)
9	62 (88.6)	66 (88.0)	60 (88.2)	65 (81.3)
10	6 (8.6)	7 (9.3)	5 (7.4)	9 (11.3)
Placental weight, mean (SD), g	553 (86)	615 (109)	561 (94)	569 (102)
pH of cord blood, mean (SD)	7.28 (0.07)	7.28 (0.06)	7.29 (0.06)	7.29 (0.09)
Anthropometry at birth, mean (SD)				
Weight, g	2969 (284)	3029 (343)	2979 (303)	3007 (328)
Height, cm	48.7 (1.5)	48.8 (1.9)	48.8 (1.7)	48.9 (1.8)
Circumference, cm				
Chest	31.4 (1.3)	31.6 (1.3)	31.4 (1.4)	31.7 (1.4)
Head	33.9 (1.2)	34.0 (1.4)	34.2 (1.1)	34.0 (1.2)

Abbreviations: CMF, cow’s milk formula; IQR, interquartile range.

^a^ Because infants did not have atopic conditions (eg, atopic dermatitis, food allergy, asthma or recurrent wheeze, or high IgE), their follow-ups ended at age 24 months.

^b^ Because infants had atopic conditions (eg, atopic dermatitis, food allergy, asthma or recurrent wheeze, or high IgE), their follow-ups were extended maximally to age 6 years.

**Figure 2.**
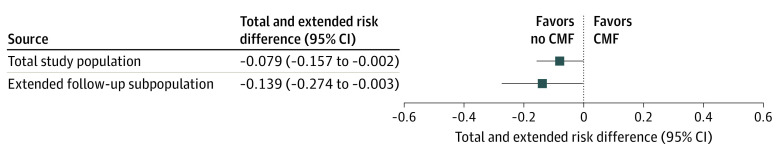
Effects of Avoiding CMF at Birth on Asthma or Recurrent Wheeze CMF indicates cow’s milk formula.

Asthma or recurrent wheeze was diagnosed in 42 infants (13.9%) at a median of 2.8 (IQR, 2.0-3.7; range, 1.2-4.6) years of age. To determine whether avoiding supplementation with CMF in the first 3 days of life decreases the risk of asthma or recurrent wheeze, the analyses were performed in the total study population and in the subpopulation with extended follow-up (Figure 2). In the overall study population, asthma or recurrent wheeze developed in 15 of 151 infants (9.9%) in the no CMF group, significantly less than in the CMF group (27 of 151 infants [17.9%]; RD, −0.079; 95% CI, −0.157 to −0.002; RR, 0.556; 95% CI, 0.308 to 1.002). Similarly, in subgroups of infants who had atopic conditions at age 2 years and continued follow-up examination to a maximum of age 6 years, the outcome was observed in 15 of 77 infants (19.5%) in the no CMF group, significantly less than in the CMF group (27 of 81 infants [33.3%]; RD, −0.139; 95%CI, −0.274 to −0.003; RR, 0.584; 95% CI, 0.338 to 1.012). The median (IQR) age at diagnosis of asthma or recurrent wheeze was 2.77 (2.02-3.73) years in the no CMF group and 2.85 (1.97-3.59) years in the CMF group, which was not significantly different.

### Levels of 25(OH)D at Age 5 and 24 Months

Both blood sampling and 25(OH)D measurements were available in 303 of 312 infants (97.1%) at age 5 months (median [IQR], 29 [17-39] ng/mL; range, 6-83 ng/mL) and in 278 of 302 infants (92.0%) at age 24 months (median [IQR] ng/mL, 21 [19-25]; range, 10-74). There was a weak association between 25(OH)D levels at age 5 months and 24 months (ρ, 0.18) (eFigure 1 in Supplement 2). There were no differences in 25(OH)D levels at age 5 and 24 months among the 4 groups (eFigure 2 in Supplement 2).

### Interaction Between 25(OH)D Levels and Avoiding CMF at Birth

**Figure 3.**
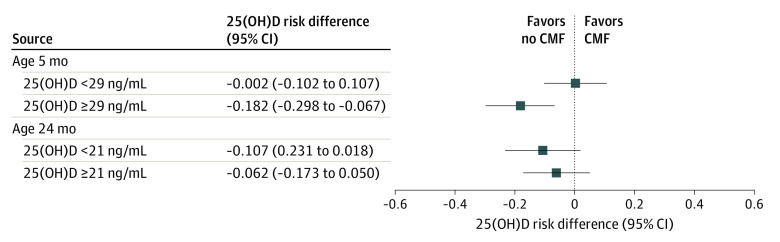
Effects of Avoiding Cow’s Milk Formula (CMF) at Birth on Asthma or Recurrent Wheeze Subgroups Stratified by 25-Hydroxyvitamin D (25[OH]D) Levels

To assess the interaction between 25(OH)D levels and the intervention on the risk of asthma or recurrent wheeze, analysis of subgroups stratified by the median 25(OH)D level was performed (Figure 3). In the subgroup of infants with 25(OH)D levels higher than the median at age 5 months, avoiding CMF at birth significantly reduced the incidence of asthma or recurrent wheeze: 5 of 78 infants (6.4%) in the no CMF group vs 17 of 69 infants (24.6%) in the CMF group (RD, −0.182; 95% CI, −0.298 to −0.067; RR, 0.260; 95% CI, 0.101-0.668). In the subgroup with lower 25(OH)D levels, avoiding CMF did not significantly reduce asthma or recurrent wheeze: 8 of 68 infants (11.8%) in the no CMF group vs 9 of 78 infants (11.5%) in the CMF group (RD, 0.002; 95%CI, −0.102 to 0.107; RR, 1.020; 95% CI, 0.417-2.496). There was a significant interaction between 25(OH)D levels and the intervention (*P* for interaction = .04). On the other hand, there was no such interaction between 25(OH)D levels at age 24 months and the intervention for the risk of asthma or recurrent wheeze.

Subgroup analysis based on tertiles of serum levels of 25(OH)D at age 5 months and 24 months was performed (eTable 1 in Supplement 2). In the subgroup of infants with middle tertile levels of 25(OH)D at 24 months of age (ie, the 103 participants with levels between 20 and 23 ng/mL), the outcome occurred in 2 participants (3.9%) in the no CMF group, which was significantly less than in the CMF group (10 [19.2%]), but the *P* value for interaction was not significant.

### Interaction Between Total IgE Levels and Avoiding CMF at Birth

**Figure 4.**
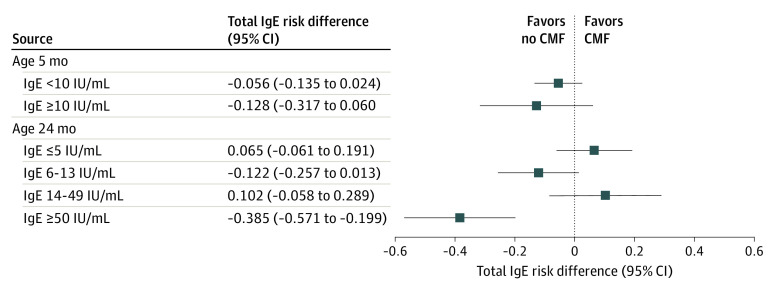
Effects of Avoiding Cow’s Milk Formula (CMF) at Birth on Asthma or Recurrent Wheeze Subgroups Stratified by Total IgE Levels

To assess the interaction between total IgE levels and the intervention on asthma or recurrent wheeze, subgroups were stratified by the median total IgE level at 5 months because almost half were not detectable at age 5 months (range, <3 to 239 IU/mL) and by quartiles of the total IgE level at age 24 months, because levels ranged broadly from less than 3 to 1250 IU/mL (Figure 4). There was no statistically significant interaction between total IgE levels at age 5 months and the intervention. However, in the subgroup of infants with the highest quartile level of total IgE at age 24 months (ie, ≥50 IU/mL), avoiding CMF at birth significantly reduced the incidence of asthma or recurrent wheeze: 2 of 38 infants (5.3%) in the no CMF group vs 14 of 32 infants (43.8%) in the CMF group (RD, −0.385; 95% CI, −0.571 to −0.199; RR, 0.120; 95% CI, 0.030-0.490; *P* for interaction = .004) but not in the other quartiles of total IgE.

### Interaction Between Allergen-Specific IgE Levels and Intervention

To assess the interaction between allergen-specific IgE levels and the intervention on asthma or recurrent wheeze, subgroups stratified by positivity, defined as having greater than the lower limit of detection (≥0.10 U_A_/mL) of each allergen-specific IgE level at age 24 months, further analysis was performed (eTable 2 in Supplement 2). In subgroups positive for IgE specific to mites, milk, and wheat (but not egg white), avoiding CMF at birth significantly reduced the risk of asthma or recurrent wheeze.

### Incidence of Asthma or Recurrent Wheeze by Duration of Adherence to the BF/EF Intervention

In the no CMF group, 40 (12.5%) mothers adhered to BF with or without EF supplementation for infants to age 5 months, 41 (7.3%) mothers switched the feeding pattern from no CMF to CMF after 14 days, and 70 (7.3%) mothers switched within 14 days after birth. The incidence of asthma or recurrent wheeze was not different among these 3 groups (eTable 3 in Supplement 2).

## Discussion

In this RCT, by avoiding exposure to CMF for at least the first 3 days of life, the risk of asthma or recurrent wheeze appeared to have been decreased compared with supplementing with CMF from the first day of life. A systematic review of 21 observational studies suggested that no BF or BF for short durations is associated with higher childhood asthma risk.^13^ In contrast, a 2002 long-term prospective cohort study^14^ demonstrated that more children who were breastfed reported current asthma at each assessment between ages 9 and 26 years than those who were not. On the other hand, a birth cohort examined in Copenhagen^15^ indicated that exclusive BF does not affect asthma or recurrent wheeze at age 7 years in at-risk children. Similarly, a large cluster RCT involving 17 046 mother-infant pairs conducted in Belarus^16^ showed that exclusive and prolonged BF did not reduce the risk of asthma at age 6.5 years.

As for the specific mechanisms behind this association, human milk oligosaccharides as well as secretory immunoglobulins, lactoferrin, and bacterial microbiota may play a role in preventing pathogenic bacterial adhesion while also providing nutrition for the microbiome.^17^ Multiple human viruses were more abundant in stool samples from infants who were exclusively fed on CMF, whereas *Bifidobacterium* and *Lactobacillus* were richer in those fed partially or fully on BF,^18^ suggesting their potential preventive effects on asthma or recurrent wheeze through gut microbiota. However, these mechanisms remain essentially unknown and further research is necessary.

A 2010 prospective cohort study of 13 019 infants^8^ demonstrated that the odds ratio for the development of IgE-mediated cow’s milk allergy in infants supplemented with CMF after 14 days of life was 19.3 compared with those supplemented within 14 days of life and thus recommended supplementation at birth with CMF to promote its tolerance. However, the authors of this study commented that because of the way their data were collected they could not exclude neonatal exposure to small quantities of CMF in the newborn nursery that was either forgotten by the mother or done without her knowledge. Other studies listed previously also cannot exclude the possibility of exposure to small amounts of CMF at birth. Thus, the results of these previous studies do not necessarily contradict those of the present trial. Moreover, about a quarter of the infants in the no CMF group adhered to BF with or without EF to age 5 months, but others switched the feeding pattern from BF with or without EF to supplementing with CMF either before or after 14 days of life. The incidence of asthma or recurrent wheeze did not differ by the duration of adherence to the no CMF intervention. This post hoc result implies that the first 3 days of life may be an important period for determining allergy or anergy.

This preventive effect on asthma or recurrent wheeze of avoiding CMF in the first 3 days of life was significant in the subgroup of infants with high 25(OH)D levels (≥29 ng/mL) at age 5 months, but not in those with low 25(OH)D levels (<29 ng/mL), suggesting an interaction between the intervention and high 25(OH)D levels. In RCTs, vitamin D supplementation during pregnancy prevented the development of asthma or recurrent wheeze in those with initial 25(OH)D levels greater than 30 ng/mL^19^ but not in the total study population.^20,21,22^

Avoiding supplementation with CMF effectively reduced the risk of asthma or recurrent wheeze only in the subgroup of infants with the highest quartile levels of total IgE (ie, ≥50 IU/mL) at age 24 months and in the subgroups of those with positive mite-, milk-, and wheat-specific IgE. Because it has been suggested that infants with elevated serum IgE levels have a predisposition to asthma at age 6 years,^23^ results of subgroup analyses implied that this intervention reduced the risk of allergic asthma^24^ but not recurrent wheeze induced by viral infection.^25,26^

### Limitations

This trial has several limitations. First, only participants with atopic conditions at their second birthdays were followed to the maximum age of 6 years. Asthma or recurrent wheeze could have developed in participants whose follow-up ended at their second birthday, which would cause bias and be the most critical issue in this study. Thus, although this was a secondary analysis of an RCT, the conclusions of this study require further investigation. Second, the sample size was originally calculated for cow’s milk–specific IgE at age 24 months. Thus, the sample size may be small for detecting risks of asthma or recurrent wheeze. In fact, the main result was marginally significant. Due to the small sample size and the weak difference, results obtained in this trial require further testing, especially from a biological plausibility or mechanistic level. Third, subgroup analyses were performed in this study, which may increase the probability of type I error due to multiple comparisons. Fourth, the present study was conducted in a single center in Japan, so that the results may not be relevant for other racial/ethnic groups and countries with different food cultures. Fifth, this trial was performed in the central area of Tokyo. Participants tended to be in a high socioeconomic class, and their children may have a high risk of atopic disease such as food allergy. Therefore, the results obtained in this trial may not be applicable to rural areas.

## Conclusions

In this study, BF with or without EF for the first 3 days or more of life appeared to decrease the risk of asthma or recurrent wheeze in young children compared with BF plus a small amount of CMF from the first day of life. These results need to be further examined.

## Notes

Supplement 1. **Trial Protocol and Statistical Analysis Plan**

Supplement 2. **eFigure 1.** Histograms of 25(OH)D levels at 5 months and 24 months of age **eFigure 2.** Comparison of 25(OH)D levels at 5 and 24 months of age among 4 groups **eTable 1.** Interaction between tertiles of 25(OH)D levels and avoiding CMF at birth **eTable 2.** Interactions between each allergen-specific IgE level at 5 months of age and the intervention **eTable 3.** Incidence of asthma or recurrent wheeze by duration of adherence to the BF/EF intervention

Supplement 3. **Data Sharing Statement**
