# Supplementary material for: Effect of Avoiding Cow's Milk Formula at Birth on Prevention of Asthma or Recurrent Wheeze Among Young Children: Extended Follow-up From the ABC Randomized Clinical Trial
Source: JAMA Netw Open. 2020 Oct 2;3(10):e2018534. doi: 10.1001/jamanetworkopen.2020.18534 (PMC7532386; doi:10.1001/jamanetworkopen.2020.18534)
Supplement: Supplement 1. — Trial Protocol and Statistical Analysis Plan [file jamanetwopen-e2018534-s001.pdf]

Trial Protocol and Statistical Analysis Plan (SAP)

Original Trial Protocol and Statistical Analysis Plan (SAP) in English p2 ~ p25
Final Trial Protocol and Statistical Analysis Plan (SAP) in English p26 ~ p50
References p51 ~ p52
Appendix 1: Table 1. Oral Food Challenge (OFC) test p53
Appendix 2: Table 2. Permutated blocks of five p54
Appendix 3: Table 2. Questionnaires of food intake p55 ~ p58
Summary of changes to the protocol approved by the ethics committee p59 ~ p61

***The statistical analysis plan is contained within the protocol files.**

Ver. 1. 2013.8.1

Trial Protocol and Statistical Analysis Plan (SAP) (original)

Title: Randomized clinical trial in preventing food allergy Breast feeding vs. breast feeding plus formula milk

ABC (Atopy prevention by Breastfeeding or Cow's milk formula)

Steering committee

Lead principal investigator

Mitsuyoshi Urashima MD, PhD, MPH
Professor of Molecular Epidemiology
The Jikei University School of Medicine
Tokyo, JAPAN

Co-investigator

Hiroshi Tachimoto MD, PhD
Associate Professor of Pediatrics
The Jikei University School of Medicine
Tokyo, JAPAN

Endpoint committee members

a. Hidetoshi Mezawa, M.D., Ph.D.,
b. Takashi Urashima, M.D., Ph.D.,
c. Daishi Hirano, M.D., Ph.D

Department of Pediatrics
The Jikei University School of Medicine
Tokyo, JAPAN

Data monitoring

a. Akio Nakashima, MD, PhD
b. Haruka Wada

Division of Molecular Epidemiology
The Jikei University School of Medicine
Tokyo, JAPAN

Safety and data monitoring committee

a. Motohiro Ebisawa, MD, PhD
b. Sakura Sato, MD, PhD
c. Kiyotake Ogura, MD, PhD

Department of Pediatrics
Sagamihara National Hospital
Kanagawa, JAPAN

Ver. 1. 2013.8.1

Protocol synopsis

Official title of the study

Randomized clinical trial in preventing food allergy Breast feeding vs. breast feeding plus formula milk

Brief title

Food allergy prevention trial

Region

Japan

Objectives

Compare atopic sensitization at 5-6 months and 2 years of age between breastfeeding with or without amino acid milk (=avoiding supplementation with cow's milk formula (CMF) for at least the first 3 days of life) and breastfeeding with CMF (=adhering to BF supplemented with CMF ≥5 ml/day from the first day of life to 1 month and ≥40ml/day after 1 month until weaning) (Efficacy)

Primary outcomes

Atopic sensitization

Cow's milk sensitization, i.e., Cow's milk-specific IgE at 24 months of age, which will be compared with other allergens sensitization, e.g., egg-white.

Key secondary outcomes

I. Immediate type of food allergy (FA) including anaphylaxis
II. Worsening of atopic dermatitis
III. Desensitization of FA
IV. Atopic sensitization alone
V. Asthmatic bronchitis
VI. Allergic rhinitis

Study design

Basic design: Parallel

Randomization: YES

Randomization unit: Individual

Blinding: Prospective, randomized, open, blinded-endpoint (PROBE) design

Control: Active

Stratification: NO

Dynamic allocation: NO

Institution consideration: NOT considered as an adjustment factor

Intervention

No. of arms: 2

Purpose of intervention: Primary prevention of food allergy (FA)

Type of intervention: Food

1. **Intervention 1**: Breastfeeding (BF) with or without amino acid-based elemental formula (EF), called the BF±EF group (if the amount of breast milk is not sufficient, EF will be added), until 5 months of age. When the mother needs to add EF of more than 150 ml/day for 3 consecutive days to BF because of a low breast milk amount, EF is switched to cow's milk formula (CMF). Thus, the offspring allocated to BF±EF can avoid CMF for at least the first 3 days of life.
2. **Intervention 2**: BF plus CMF (≥5 ml/day from the first day of life to 1 month and ≥40 ml/day after 1 month until weaning), called the BF+CMF group, until 5 months of age or starting solid food.

Participants

Age: Neonates at birth

Gender: Male and female

Key inclusion criteria

1. Be in a high-risk group for atopic sensitization: at least one of the father, mother, brothers,

or sisters of infants has current and/or past atopic diseases (asthma, atopic dermatitis, food allergy, allergic rhinitis, hay fever)

2. Obtain written informed consent.

   a. Participants who understand randomization into two groups: BF with or without amino acid milk and BF with cow's formula milk.

   b. Blood examination at 5-6 months and 2 years of age is required.

3. Be available to visit the outpatient clinic at 1 month, 2 months, 3 months, 4 months, 5 months, 7~8 months, 9~10 months, 12 months, 14~15 months, 18 months, and 24 months of age.

Key exclusion criteria

1. Less than 36 weeks of gestational age at birth or birth weight less than 2000 g.
2. Having serious congenital anomalies.
3. Being a triplet or more; twins may participate.
4. Abnormal delivery such as asphyxia (Apgar score 6 or less)

Target sample size

Total, n = 300;

BF±EF group, n = 150;

BF+CMF group, n = 150.

Location of the trial

Tokyo, Japan

a. Patient care: Outpatient clinic of Jikei University Hospital

b. Data monitoring and analysis: Division of Molecular Epidemiology, Jikei University School of Medicine

Study period

Accrual, if the number of randomized participants reaches >300, then entry is stopped.

The trial ends when the last participant is followed up after the 2-year-old birthday.

Follow-up

Participating parent(s) are interviewed, and their children are examined from 1 to 24 months of age at the outpatient clinic of Jikei University Hospital. All infants undergo blood examinations to measure serum levels of total IgE and antigen-specific IgE at 5 and 24 months of age. Unscheduled visits and blood examinations are performed at the clinic when a participating infant has atopic symptoms, particularly when FA is suspected.

Statistical analysis

I. Primary outcome: Cow's milk-specific IgE

A) Antigen-specific IgE ≥0·35 U_A/ml at 24 months of age as a dichotomous variable, risk ratio (RR) and 95% confidence intervals (95% CI)

B) Serum levels of cow's milk-specific IgE as a continuous variable at 5 and 24 months of age: Mann-Whitney test between the comparative groups.

II. Secondary outcomes

Clinical food allergy: RR, and 95% CI are used to evaluate the secondary outcomes.

Ethics

1. Private information is carefully protected.
2. All personal information about the participants is rendered innominate in a linkable fashion at Jikei University Hospital.
3. Liability insurance is obtained to compensate participants for side effects of interventions.

Funding

The Jikei University School of Medicine

Background

The prevalence of food allergy (FA) has been growing over the last 25 years. Especially, the frequency of anaphylaxis, which may be fatal, has increased 5 times since 1990 in developed countries.[1-3] In 2000, the American Academy of Pediatrics (AAP) made the following recommendations: Infants at high risk for developing allergy, identified by a strong (one or both parents, sibling) family history of allergy, may benefit from the following: (1) Breastfeeding (BF) mothers should continue BF for the first year of life or longer. During this time, for infants at risk, hypoallergenic formulas can be used to supplement BF; (2) Mothers should eliminate peanuts and tree nuts (e.g., almonds, walnuts, etc.) and consider eliminating eggs, cow's milk, fish, and perhaps other foods from their diets while nursing; (3) Solid foods should not be introduced into the diet of high-risk infants until 6 months of age, with dairy products delayed until 1 year, eggs until 2 years, and peanuts, nuts, and fish until 3 years of age.[4] However, in 2008, AAP revised the recommendations as follows: (1) For infants at high risk of developing atopic disease, there is evidence that exclusive BF for at least 4 months compared with feeding intact CMF decreases the cumulative incidence of atopic dermatitis and cow's milk allergy (CMA) in the first 2 years of life. Comparative studies of the various hydrolyzed formulas have also indicated that not all formulas have the same protective benefit. Extensively hydrolyzed formulas may be more effective than partially hydrolyzed formulas for the prevention of atopic disease; (2) At the present time, antigen avoidance during lactation does not prevent atopic disease, although more data are needed to substantiate this conclusion; (3) Although solid foods should not be introduced before 4 to 6 months of age, there is no current convincing evidence that delaying their introduction beyond this period has a significant protective effect on the development of atopic disease regardless of whether infants are fed cow's milk protein formula or human milk.[5]

Although WHO recommends BF for the first 6 months of life to improve childhood morbidity and mortality,[6] supplementation of BF with cow's milk formula (CMF) is quite common in many countries including Japan. In contrast to WHO's recommendation, a population-based prospective cohort study in Israel demonstrated that the frequency of IgE-mediated CMA can be dramatically lowered if infants start receiving regular CMF within the first 14 days of

life (0.05%) compared with beginning CMF between 105 and 194 days (1.75%). They thus recommended CMF supplementation at birth to promote oral tolerance to cow's milk.[7] Although such studies do not eliminate the possibility of reverse causality as an explanation for this finding (high-risk infants with eczema are deliberately breast-fed longer) due to the nature of observational studies, they raise the question as to whether exposure to CMF as a supplement to BF within 14 days of life may play a role in preventing allergic disease. Indeed, the German Infant Nutritional Intervention Study Group disagreed because they could not reproduce the risk reduction by early exposure to CMF in post-hoc analysis of their previous randomized controlled trial.[8]

Sometimes during the first 3 days of life, the amounts of breast milk are small or postpartum mothers are too tired to breast feed. Consequently, newborn infants are quite often supplied CMF in addition to BF during hospital admission. Indeed, many Japanese maternity wards basically recommend BF, but allow mothers or nurses to help by supplying CMF as a supplement to BF after feeding colostrum, e.g., 6 to 10 hours after birth. In addition to the controversy regarding CMF supplementation before or after day 14, whether supplementary feeding with CMF during the first 3 days of life is protective,[9] neutral,[10] or rather enhances[11] risks of atopic diseases is also controversial, as shown in randomized controlled trials. In 1988, a cohort study in Denmark showed that all 39 CMAs developed in 1,539 infants who had been supplied CMF during the first 3 days of life, whether breast fed or not, after discharge from the maternity hospital, whereas no CMAs were observed in 210 infants who had not been supplied CMF.[12] In the discussion of the cohort study in Israel, the authors stated: "Because of the way our data were collected, we cannot exclude neonatal exposure to small quantities of CMP formula in the newborn nursery either forgotten by the mother or done without her knowledge. However, the role of a brief intermittent early exposure to milk in the neonatal unit is controversial[10,11] and might have a low effect, if any, on the development of atopy. Accordingly, we found it appropriate not to consider such intermittent exposures to CMP in this study".

Hypotheses

1. Does avoiding exposure to CMF for the first 3 days of life prevent development of FA later?

   This is the main hypothesis of this study and was explained in the ‘Background’.

   Many Japanese maternity wards encourage BF, but allow mothers or nurses to supplement BF with CMF, e.g., approximately 6 to 10 hours after birth or even earlier, based on maternal preferences, but not based on clinical evidence. However, more than 20 to 30 years ago, sugar water was given instead of CMF supplement at birth. Thus, we hypothesized that early exposure to CMF at birth may be associated with the recent increase in children with FA. Therefore, a randomized clinical trial (RCT) was conducted to assess whether the risk of CMF sensitization and FA is decreased by either avoiding or supplementing CMF at birth.

2. Vitamin D hypotheses

   Refer to the review article by Dr. G. Lack.[13]

   The vitamin D hypothesis takes two forms: the vitamin D excess hypothesis argues that increases in vitamin D levels have led to increased allergies. The vitamin D deficiency hypothesis argues the opposite.

Objectives

Specific Aim 1: To compare atopic sensitization at 5-6 months and 2 years of age between BF with or without amino acid milk and BF with CMF.

Specific Aim 2: Subgroup analysis based on serum levels of 25-hydroxyvitamin D (25OHD) at 5 months, e.g., tertile, may be performed.

Study design

Randomized, controlled, parallel two-group trial at a single institution

Pregnant women during the third trimester are randomly assigned to either BF with or without amino acid-based EF, called the BF±EF group, or BF plus CMF (≥5 ml/day from the first day of life to 1 month and ≥40 ml/day after 1 month until weaning), called the BF+CMF group, in a 1:1 ratio. Infants are followed until they are at least 2 years of age. Follow-up is extended 1 year if participating infants have FA. When the mother needs to add more than 150 ml/day EF for 3 consecutive days to BF because of a low amount of breast milk, EF is switched to CMF. Thus, the offspring allocated to BF±EF can avoid CMF for at least the first 3 days of life.

Subgroup

1. Tertiles of 25(OH)D levels at 5 months of age

Measure 25OHD levels in the serum obtained at 5 months of age.

Research Implementation System

I. Clinical examination

a. Pediatrician: Dr. Mitsuyoshi Urashima: Enrollment and participants' clinical examination before allergic signs. If participating infants have any allergic signs, i.e., food-specific IgE ≥0.35 U_A/ml blood sampling at 5 months of age, then they are referred to Dr. Tachimoto to diagnose atopic diseases and for follow-up.
   At the outpatient clinic of The Maternal and Child Center in Jikei University Hospital
b. Pediatrician: Dr. Hiroshi Tachimoto: Clinical examination after allergic signs and diagnosis of atopic diseases with or without the oral food challenge (OFC) test, management, and follow-up.
   At the outpatient clinic of Pediatrics in Jikei University Hospital

II. Endpoint committee

Among the following five members who do not know the allocation group and will not examine these participating infants at outpatient clinics, if ≥2 recognize the outcome as positive, the outcomes are finally determined to be positive.

1. Hidetoshi Mezawa, M.D., Ph.D.
2. Takashi Urashima, M.D., Ph. D.
3. Daishi Hirano, M.D., Ph.D.

Department of Pediatrics
The Jikei University School of Medicine
Tokyo, Japan

What the committee is expected to determine.

These five members determine whether the infant has had an episode of FA or not.

0. No FA
1. Immediate allergic reaction
2. Anaphylaxis

For 1 (and 2) above, they determine the status at the second birthday by reviewing detailed episodes, results of the OFC test, and levels of food-specific IgE.

1. Sustained unresponsiveness = outgrow
   Cow’s milk allergy, the infant can drink raw milk ≥50 ml
   Egg allergy, the infant can eat baked egg ≥30g,
   Wheat allergy, the infant can eat udon noodle ≥15g
2. Probable unresponsiveness, e.g., in the process of increasing dose of allergenic food, but not yet reach to predefined dose of unresponsiveness mentioned above.
3. Not yet unresponsive, when the infant eats, he or she still reacts to the allergenic food.
4. Not sure because the infant has not consumed the food allergen

The infants are still state of either 2, 3, or 4 above, they are considered not yet outgrow = remain food allergy.

III. Data monitoring

A) Randomization using a block of four by Dr. Akio Nakashima who does not interview participating families and does not examine their infants.

B) Data monitoring

If the frequency of adverse events deviates in either intervention group, the committee reports the data to the Safety and Data Monitoring Committee as well as the lead investigator.

a. Akio Nakashima, M.D., Ph.D. (physician and nephrologist)

b. Ms. Haruka Wada (secretary at Molecular Epidemiology)

Division of Molecular Epidemiology
The Jikei University School of Medicine
Tokyo, Japan

IV. Safety and Data Monitoring Committee

a. Judging from data of interim analysis, this committee may recommend stopping or continuing the trial.

b. Responding to a report of adverse events, the committee may recommend stopping the trial or allowing the trial to continue.

① Motohiro Ebisawa, M.D., Ph.D.

② Sakura Sato, M.D., Ph.D.

③ Kiyotake Ogura, M.D., Ph.D.

Department of Pediatrics
Sagamihara National Hospital
Kanagawa, Japan

Participants

Inclusion criteria

1. Be in a high-risk group for atopic sensitization: at least one of the father, mother,

brothers, or sisters of infants has atopic diseases (asthma, atopic dermatitis, FA, allergic rhinitis, hay fever)

2. Obtained written informed consent.

   a. Participants who understand randomization into two groups: BF with or without amino acid milk and BF with CMF.

   b. Blood examinations at 5-6 months and 2 years of age are required.

3. Be available to visit the outpatient clinic at 1 month, 2 months, 3 months, 4 months, 5 months, 7~8 months, 9~10 months, 12 months, 14~15 months, 18 months, and 24 months of age.

Exclusion criteria

1. Less than 36 weeks of gestational age at birth or birth weight less than 2000 g.
2. Having serious congenital anomalies.
3. Being a triplet or more: twins may participate.
4. Abnormal delivery such as asphyxia (Apgar score 6 or less at 5 minutes after birth)

Interventions

1. BF±EF

Mothers in this group are asked to maintain exclusive BF. However, if the amount of breast milk is not sufficient, then EF may be added until starting solid food. If the mother needs to supply EF more than 150 ml/day for 3 consecutive days in addition to BF because of a low amount of breast milk, EF is switched to CMF. Thus, the offspring allocated to BF±EF can avoid CMF for at least the first 3 days of life.

2. BF+CMF

Mothers in this group are asked to maintain exclusive BF, but are also asked to simultaneously supply a small amount of CMF ≥5 ml/day from the first day of life until 1 month, and ≥40 ml/day after 1 month until weaning. When infants are born after 18:00, CMF may be added on the 2nd day.

Both CMF (Hohoemi®) and EF (Meiji Elemental formula®) are purchased from Meiji Holdings Co. Ltd. (Chuo-ku, Tokyo, Japan).

EF (Meiji Elemental formula®)

For infants with FA, total proteins are replaced with refined amino acids. However, other fats, carbohydrates, vitamins, and minerals are basically the same as in CMF. Energy is 66 kcal/100 ml (17%) and 391 kcal/100 g powder.

Blinding to allocation group

a. Participating family: Not blinded

b. Dr. M.U.: Not blinded, because he needs to confirm compliance with the intervention by interviewing participating families at the outpatient clinic in the Maternal and Child Center of Jikei University Hospital. When an infant has food-specific IgE ≥0.10 U_A/ml at 5 months blood test, the infant is referred to Dr. H.T. at the outpatient clinic of Pediatrics without informing Dr. H.T. about the allocation group.

c. Dr. H.T.: Blinded, who is a specialist in pediatric allergic diseases, diagnoses FA, without knowing the allocation group.

d. Endpoint committee: Blinded and no contact with the participants.

Outcome Measures

Primary outcome

1. **Atopic sensitization:**

   The primary outcome was atopic sensitization to cow's milk protein, i.e., serum levels of cow's milk-specific IgE (CM-IgE) ≥0·35 UA/ml at 24 months of age. Serum levels of CM-IgE at 5 and 24 months of age as a continuous variable were used as reference values for primary outcome. Other atopic sensitization to egg white and wheat ≥0·35 UA/ml at 24 months of age were also evaluated to be compared with cow's milk sensitization. Outsourced for measuring with ImmunoCAP Specific IgE® (Thermo Fisher Diagnostics K.K., Minato-ku, Tokyo, Japan) to SRL Inc. and compared as both continuous and dichotomous (CM-IgE: ≥0.35 UA/ml) variable between BF±EF and BF+CMF group.

Secondary outcomes

1. Immediate type of FA

Presenting with one organ symptom of skin (urticaria, angioedema, erythema, pruritus, periorbital edema, etc.), respiratory (dry staccato cough, hoarseness, wheezing, etc.), gastrointestinal (nausea, vomiting, colicky abdominal pain, etc.), or circulatory (tachycardia, hypotension, loss of consciousness, etc.) problems triggered by the food ingestion that appeared within a few hours, in combination with blood examination measuring serum levels of the suspected food-specific IgE: ≥0.35 U_A/ml by means of a fluorescence enzyme immunoassay.

In this trial, symptoms triggered by skin contact directly with food or indirectly with food-contaminated hands are not included.

The OFC test (Appendix 1) is performed around 1 year of age for infants who are sensitized to certain foods but for whom clinical reactivity after ingestion of the food allergen is unknown. Thus, immediate allergic reaction is a food-specific IgE: ≥0.35 U_A/ml plus either a FA episode or positive OFC test. FA is re-evaluated regarding whether the infant could eat the causative food allergen without an allergic reaction at the 2-year-old birthday.

2. Anaphylactic type of FA

When symptoms are derived from multiple organs, e.g., urticaria and wheezing, the condition is defined as an anaphylactic reaction.

3. Worsening of atopic dermatitis

Check the SCORAD index[14] at the 1- and 3-month check-ups.

4. Desensitization of food allergy (= unresponsiveness)

If infants, who had been once diagnosed as immediate type of FA especially with cow's milk allergy, egg allergy, and wheat allergy, can eat the allergenic food (raw milk ≥50 ml, baked egg 30g, udon noodle 15g) without allergic signs by 2nd birthday, then they are defined as 'sustained unresponsiveness = outgrow' to the food

allergen in this trial.

5. **Atopic sensitization alone**

Although serum levels of food-specific IgE are positive (≥0.35 U_A/ml), the infant never had allergic signs and symptoms even by eating the allergenic food.

6. **Asthmatic bronchitis**

A stringent index that includes frequent wheezing during the first 3 years of life and either one major risk factor (parental history of asthma or eczema) or two of three minor risk factors (eosinophilia, wheezing without colds, and allergic rhinitis).[15]

Major Criteria

1. Parental asthma†
2. Eczema diagnosed by a medical doctor‡
3. Sensitization with elevated mite- or house dust-specific IgE ≥0.10 U_A/ml

Minor Criteria

1. Allergic rhinitis diagnosed by a medical doctor§
2. Wheezing apart from colds
3. Eosinophilia (≥4%)

7. **Allergic rhinitis**

a. Hay fever, e.g., Sugi pollen allergy: sugi pollen-specific IgE ≥0.10 U_A/ml

b. Allergic rhinitis: Rhinitis signs and symptoms for 1 month and mite- or house dust-specific IgE ≥0.10 U_A/ml

Safety outcomes

The following are obtained from an interview at the outpatient clinic just after the 2-year-old birthday.

1. Admission for any reason
2. Emergency room visit for any reason
3. Growth retardation: Height and weight at 2-year-old birthday

Flow of participants

1. Informed consent and registration

a. Pregnant women and their husbands are informed about the trial in detail at a childbirth class during late pregnancy through a talk by Dr. M. Urashima and the informed consent document. Written, informed consent is sent to the data monitoring center at the Molecular Epidemiology Division of Jikei University School of Medicine.

b. Current or previous family history of atopic diseases (asthma, atopic dermatitis, FA, allergic rhinitis, hay fever) is obtained through questionnaires as well as parental demographic information such as maternal age and body weight/height. Only pregnant women who are confirmed to have a family history of atopic diseases are included.

c. Food questionnaires of mothers (Appendix 3) are taken during the third trimester of pregnancy and at 1, 3, and 5 months of age after birth.

2. At the maternity ward of Jikei University Hospital

a. Some included pregnant women are excluded after delivery, due to, e.g., cleft palate.

b. Perinatal information, e.g., gestational weeks and birth weight, are taken from charts.

c. Nursing staff is informed of the participants' names and to which group they are allocated.

d. Mothers are informed to which group they belong after delivery; thus, mothers are not blinded to the allocation group.

e. Mothers belonging to the BF+CMF group are asked to supplement BF with CMF at least 5 ml from the first day of delivery. However, if the delivery time is after 18:00, they are allowed to supply CMF in addition to BF starting at midnight of the 2nd day.

f. Mothers belonging to the BF±EF group may add amino acid-based EF when they feel that BF is not enough. If the mother adds more than 150 ml/day EF to BF for 3 consecutive days, EF is switched to CMF. Thus, offspring allocated to BF±EF can avoid CMF for at least the first 3 days of life.

g. Nurses at the maternity ward are allowed to feed babies instead of their mothers.

3. At the outpatient clinic in the Maternal and Child Center of Jikei University Hospital

a. Participating parent(s) are interviewed, and their children are examined at least seven times from 1, (2,) 3, (4,) 5-6, (7-8,) 9-10, 12, (14-15,) 18, and 24 months of age at the outpatient clinic of Jikei University Hospital by a study pediatrician (M.U.) who is not blinded to the allocation group.

b. If infants have exanthema, the pediatrician provides their parents clear guidance about aggressive skin care such as how to wash their face and body using soap and a shower by showing them a video.

c. Blood is sampled from all infants at 5-6 and 24 months.

4. At the outpatient clinic in Pediatrics of Jikei University Hospital

In case of at least one antigen-specific IgE is 0.10 IU_A/ml or more, the infant is referred to a study specialist of pediatric allergy (H.T.) at the same Jikei University Hospital who does not know the infant’s allocation group.

5. Pediatrics Ward of Jikei University Hospital

The OFC test is performed at admission (Appendix 1) by pediatricians who are blinded to the allocation group.

6. Efforts to reduce loss to follow-up

a. Using email, we try to contact participants who move to distant places regarding whether their infants have FA and make efforts to reduce the number of infants lost to follow-up, from whom IgE data are not obtained at 5 or 24 months.

b. If participants do not visit the outpatient clinic after their 2-year-old birthday and we receive no responses to the email, they are considered lost to follow-up.

7. Blood sampling

All infants undergo blood examinations to measure serum total and antigen-specific IgE.

a. At the 5- to 6-month check-up prior to first solid food introduction: Egg white, cow’s milk, wheat, mites.

b. At 2 years of age: Egg white, cow’s milk, wheat, mites, Japanese cedar pollen, dog dandruff, cat dandruff.

c. Unscheduled visits are made to the clinic when participating infants have atopic symptoms, particularly suspicious of FA. e.g., exanthema just after eating an egg dish.

8. Randomization

a. EF and CMF

Both CMF (Hohoemi®) and EF (Meiji Elemental formula®) are purchased from Meiji Holdings Co. Ltd. (Chuo-ku, Tokyo, Japan).

b. Randomization

Pregnant women that meet the inclusion criteria are randomly assigned to either the BF+CMF or BF±EF group according to an identification number generated by computer based on permutated blocks of four that are determined beforehand (Appendix 2). Staff members at the maternity ward are informed of which intervention applies to each pregnant participant before hospitalization for childbirth.

c. Blinding

According to the nature of the intervention, we do not use double blinding. However, we try to minimize bias using the following strategies:

Primary outcome: Cow’s milk-specific IgE as an objective indicator.

Secondary outcomes: determine by prospective, randomized, open, blinded-endpoint (PROBE), determined by the endpoint committee members who do not examine the infants or are blinded to the allocation group’.

9. Compliance with CMF or EF

1. At the maternity ward, every day’s CMF or EF intake is monitored by each

participant (mother). The nurse may help with the intervention and monitoring.

2. At the outpatient clinic, mothers are asked whether they are using CMF, EF, or exclusive BF.

10. Monitoring of secondary outcomes

a. Scheduled

At either the Maternal and Child Center or Pediatrics of Jikei University Hospital, participating infants are examined frequently.

b. Unscheduled

If the infants have any allergic signs, e.g., urticaria after eating allergenic food, they are advised to call Jikei University Hospital. H.T. or other pediatricians try to examine the infants on the day or next day and perform a blood examination.

Statistical analysis

1. Sample size

300

2. Sample size calculation

300 pregnant women and their offspring

We hypothesized that cow's milk sensitization at 2 years of age, i.e., cow's milk-specific IgE ≥0.35 U_A/ml, would be present in 10% of one group of infants and 25% of the other group, with a type I error (two-sided) of 5% and a power of 90%, on the assumption of 3% loss to follow-up. Therefore, 1:1 divided samples of approximately 300 participants were calculated as sufficient to detect a significant difference.

. sampsi .1 .25

Estimated sample size for two-sample comparison of proportions

Test Ho: p1 = p2, where p1 is the proportion in population 1

and p2 is the proportion in population 2

Assumptions:

alpha = 0.0500 (two-sided)
power = 0.9000
p1 = 0.1000
p2 = 0.2500
n2/n1 = 1.00

Estimated required sample sizes:

n1 = 146
n2 = 146

n = 292

Assuming 3% loss to follow-up, 300 are needed to enroll.

3. Study period

We assume that 100 pregnant women per year will participate in this trial. Therefore, the accrual period will be 3 years. The last infant is followed up 2 years later. Thus, a total of 5 years is predicted to be needed to accomplish this trial.

4. Interim analysis

Interim analyses are planned two times at reaching 100 and 200 participating infants who had their second birthday. The P value for significance at the interim analysis is set as <0.001 according to the Peto's stopping boundaries.

5. Planned methods of analysis

Outcomes are analyzed by intention-to-treat analyses between the two comparative groups.

a. Serum levels of cow's milk-specific IgE at 5 months and 24 months of age as a continuous variable are compared using Mann-Whitney test.

b. Risk of cow's milk-specific IgE ≥0.35 U_A/ml will be analyzed with a 2x2 table with the chi-square test and represented by the risk ratio (RR), risk difference (RD) and 95% confidence interval (95% CI).
c. Secondary outcomes will be compared with RR, RD, and 95% CI.
d. Safety outcomes will be compared with RR, RD and 95% CI or Student's t test.
e. Subgroup analysis will be done according to levels of 25(OH)D.
f. All reported P values will be two-sided.
g. Values with $P < 0.05$ will be considered statistically significant.
h. All data will be statistically analyzed using Stata 14.0 (StataCorp LP., College Station, TX, USA).

6. Subgroup analyses

To clarify whether BF±EF or BF+CMF significantly affects the subgroups of 25(OH)D (tertile), P for interaction ($P_{interaction}$) is computed by creating multiplicative variables. The results of these analyses are not corrected for multiple comparisons.

Safety

Many Japanese maternity wards recommend BF, but allow mothers or nurses to help by supplying CMF to supplement BF after feeding colostrum, e.g., 6 to 10 hours after birth. This is a food intervention trial. Thus, basically no safety concerns exist. However, the following are compared.

a. Admission due to any disease
b. Emergency visit or admission for any reason
c. Body height and weight at 24 months of age

Early withdrawal

Unknown serious adverse events.

Compensation

Mitsui Sumitomo Insurance Fire provides liability insurance (NB41027726) for the principal investigator, to compensate for costs of treating disability as a result of side

effects caused by consuming milk.

The maximum amount of compensation is 100,000,000 yen per person and 300,000,000 yen per trial.

Consideration concerning the protection of human rights and privacy

1. Written informed consent is obtained after sufficient explanation.
2. Participants can withdraw from the trial after providing written, informed consent.
3. Withdrawal is not considered a disadvantage for participants.
4. Private information is exchanged with study ID at the Jikei University School of Medicine; therefore, private information cannot leak from there (linkable anonymizing).

Publication of research results

We aim to publish the results of this trial in a scientific journal or present them at academic conferences. Personal information will be carefully concealed when we present the results.

Research funds

1) The Jikei University School of Medicine

Attribution of intellectual property rights

If intellectual property rights such as patent rights become relevant, such rights will be attributable to the investigator.

2017.12.25

Trial Protocol and Statistical Analysis Plan (SAP) (final)

Title: Randomized clinical trial in preventing food allergy Breast feeding vs. breast feeding plus formula milk

ABC (Atopy prevention by Breastfeeding or Cow's milk formula)

Steering committee

Lead principal investigator

Mitsuyoshi Urashima MD, PhD, MPH
Professor of Molecular Epidemiology
The Jikei University School of Medicine
Tokyo, JAPAN

Co-investigator

Hiroshi Tachimoto MD, PhD
Associate Professor of Pediatrics
The Jikei University School of Medicine
Tokyo, JAPAN

Endpoint committee members

a. Hidetoshi Mezawa, M.D., Ph.D.,
b. Takashi Urashima, M.D
c. Daishi Hirano, M.D., Ph.D
d. Mai Okuyama, M.D
e. Noriko Gocho, M.D.

Department of Pediatrics
The Jikei University School of Medicine
Tokyo, JAPAN

Data monitoring

a. Akio Nakashima, MD, PhD
b. Haruka Wada

Division of Molecular Epidemiology
The Jikei University School of Medicine
Tokyo, JAPAN

Safety and data monitoring committee

a. Motohiro Ebisawa, MD, PhD

b. Sakura Sato, MD, PhD

c. Kiyotake Ogura, MD, PhD

Department of Pediatrics
Sagamihara National Hospital
Kanagawa, JAPAN

Ver. 1. 2013.8.1
Ver. 2. 2016.7.22
Ver. 3. 2017.12.25

Protocol synopsis

Official title of the study

Randomized clinical trial in preventing food allergy Breast feeding vs. breast feeding plus formula milk

Brief title

Food allergy prevention trial

Region

Japan

Objectives

Compare atopic sensitization at 5-6 months and 2 years of age between breastfeeding with or without amino acid milk (=avoiding supplementation with cow's milk formula (CMF) for at least the first 3 days of life) and breastfeeding with CMF (=adhering to BF supplemented with CMF ≥5 ml/day from the first day of life to 1 month and ≥40ml/day after 1 month until weaning) (Efficacy)

Primary outcomes

Atopic sensitization

Cow's milk sensitization, i.e., Cow's milk-specific IgE at 24 months of age, which will be compared with other allergens sensitization, e.g., egg-white.

Secondary outcomes

I. Immediate type of food allergy (FA) including anaphylaxis
II. Desensitization of FA (= sustained unresponsiveness, or outgrow)

Exploratory outcomes

I. Atopic dermatitis
II. Asthmatic bronchitis

III. Sensitization to Japanese cedar pollen

IV. Allergic rhinitis

Study design

Basic design: Parallel

Randomization: YES

Randomization unit: Individual

Blinding: Prospective, randomized, open, blinded-endpoint (PROBE) design

Control: Active

Stratification: NO

Dynamic allocation: NO

Institution consideration: NOT considered as an adjustment factor

Intervention

No. of arms: 2

Purpose of intervention: Primary prevention of FA

Type of intervention: Food

1. **Intervention 1**: Breastfeeding (BF) with or without amino acid-based elemental formula (EF), called the BF±EF group (if the amount of breast milk is not sufficient, EF will be added), until starting solid food. When the mother needs to add EF of more than 150 ml/day for 3 consecutive days to BF because of a low breast milk amount, EF is switched to cow's milk formula (CMF). Thus, the offspring allocated to BF±EF can avoid CMF for at least the first 3 days of life.
3. **Intervention 2**: BF plus CMF (≥5 ml/day from the first day of life to 1 month and ≥40 ml/day after 1 month until weaning), called the BF+CMF group, until 5 months of age or starting solid food.

Participants

Age: Neonates at birth

Gender: Male and female

Key inclusion criteria

1. Be in a high-risk group for atopic sensitization: at least one of the father, mother, brothers, or sisters of infants has current and/or past atopic diseases (asthma, atopic dermatitis, food allergy, allergic rhinitis, hay fever)
2. Obtain written informed consent.
   a. Participants who understand randomization into two groups: BF with or without amino acid milk and BF with cow's formula milk.
   b. Blood examination at 5-6 months and 2 years of age is required.
3. Be available to visit the outpatient clinic at 1 month, 2 months, 3 months, 4 months, 5 months, 7~8 months, 9~10 months, 12 months, 14~15 months, 18 months, and 24 months of age.

Key exclusion criteria

1. Less than 36 weeks of gestational age at birth or birth weight less than 2000 g.
2. Having serious congenital anomalies.
3. Being a triplet or more; twins may participate.
4. Abnormal delivery such as asphyxia (Apgar score 6 or less)

Target sample size

Total, n = 300;

BF±EF group, n = 150;

BF+CMF group, n = 150.

Location of the trial

Tokyo, Japan

a. Patient care: Outpatient clinic of Jikei University Hospital
b. Data monitoring and analysis: Division of Molecular Epidemiology, Jikei University School of Medicine

Study period

Accrual, if the number of randomized participants reaches >300, then entry is stopped. The trial ends when the last participant is followed up after the 2-year-old birthday.

Follow-up

Participating parent(s) are interviewed, and their children are examined from 1 to 24 months of age at the outpatient clinic of Jikei University Hospital. All infants undergo blood examinations to measure serum levels of total IgE and antigen-specific IgE at 5 and 24 months of age. Unscheduled visits and blood examinations are performed at the clinic when a participating infant has atopic symptoms, particularly when FA is suspected.

Statistical analysis

I. Primary outcome: Cow's milk-specific IgE

A) Antigen-specific IgE ≥0·35 U_A/ml at 24 months of age as a dichotomous variable, risk ratio (RR) and 95% confidence intervals (95% CI)

B) Serum levels of cow's milk-specific IgE as a continuous variable at 5 and 24 months of age: Mann-Whitney test between the comparative groups.

II. Secondary outcomes

Clinical food allergy: RR, and 95% CI are used to evaluate the secondary outcomes.

Ethics

1. Private information is carefully protected.
2. All personal information about the participants is rendered innominate in a linkable fashion at Jikei University Hospital.
3. Liability insurance is obtained to compensate participants for side effects of interventions.

Funding

1. JSPS KAKENHI Grant Number JP16K09074
2. The Practical Research Project for Allergic Disease and Immunology of the Japan Agency for Medical Research and Development, AMED (15ek0410019h0101)

3. Dairy Products Health Science Council and Japan Dairy Association

Background

The prevalence of food allergy (FA) has been growing over the last 25 years. Especially, the frequency of anaphylaxis, which may be fatal, has increased 5 times since 1990 in developed countries.[1-3] In 2000, the American Academy of Pediatrics (AAP) made the following recommendations: Infants at high risk for developing allergy, identified by a strong (one or both parents, sibling) family history of allergy, may benefit from the following: (1) Breastfeeding (BF) mothers should continue BF for the first year of life or longer. During this time, for infants at risk, hypoallergenic formulas can be used to supplement BF; (2) Mothers should eliminate peanuts and tree nuts (e.g., almonds, walnuts, etc.) and consider eliminating eggs, cow's milk, fish, and perhaps other foods from their diets while nursing; (3) Solid foods should not be introduced into the diet of high-risk infants until 6 months of age, with dairy products delayed until 1 year, eggs until 2 years, and peanuts, nuts, and fish until 3 years of age.[4] However, in 2008, AAP revised the recommendations as follows: (1) For infants at high risk of developing atopic disease, there is evidence that exclusive BF for at least 4 months compared with feeding intact CMF decreases the cumulative incidence of atopic dermatitis and cow’s milk allergy (CMA) in the first 2 years of life. Comparative studies of the various hydrolyzed formulas have also indicated that not all formulas have the same protective benefit. Extensively hydrolyzed formulas may be more effective than partially hydrolyzed formulas for the prevention of atopic disease; (2) At the present time, antigen avoidance during lactation does not prevent atopic disease, although more data are needed to substantiate this conclusion; (3) Although solid foods should not be introduced before 4 to 6 months of age, there is no current convincing evidence that delaying their introduction beyond this period has a significant protective effect on the development of atopic disease regardless of whether infants are fed cow’s milk protein formula or human milk.[5]

Although WHO recommends BF for the first 6 months of life to improve childhood morbidity and mortality,[6] supplementation of BF with cow’s milk formula (CMF) is quite common in many countries including Japan. In contrast to WHO’s recommendation, a population-based prospective cohort study in Israel demonstrated that the frequency of IgE-mediated CMA can be dramatically lowered if infants start receiving regular CMF within the first 14 days of

life (0.05%) compared with beginning CMF between 105 and 194 days (1.75%). They thus recommended CMF supplementation at birth to promote oral tolerance to cow's milk.[7] Although such studies do not eliminate the possibility of reverse causality as an explanation for this finding (high-risk infants with eczema are deliberately breast-fed longer) due to the nature of observational studies, they raise the question as to whether exposure to CMF as a supplement to BF within 14 days of life may play a role in preventing allergic disease. Indeed, the German Infant Nutritional Intervention Study Group disagreed because they could not reproduce the risk reduction by early exposure to CMF in post-hoc analysis of their previous randomized controlled trial.[8]

Sometimes during the first 3 days of life, the amounts of breast milk are small or postpartum mothers are too tired to breast feed. Consequently, newborn infants are quite often supplied CMF in addition to BF during hospital admission. Indeed, many Japanese maternity wards basically recommend BF, but allow mothers or nurses to help by supplying CMF as a supplement to BF after feeding colostrum, e.g., 6 to 10 hours after birth. In addition to the controversy regarding CMF supplementation before or after day 14, whether supplementary feeding with CMF during the first 3 days of life is protective,[9] neutral,[10] or rather enhances[11] risks of atopic diseases is also controversial, as shown in randomized controlled trials. In 1988, a cohort study in Denmark showed that all 39 CMAs developed in 1,539 infants who had been supplied CMF during the first 3 days of life, whether breast fed or not, after discharge from the maternity hospital, whereas no CMAs were observed in 210 infants who had not been supplied CMF.[12] In the discussion of the cohort study in Israel, the authors stated: "Because of the way our data were collected, we cannot exclude neonatal exposure to small quantities of CMP formula in the newborn nursery either forgotten by the mother or done without her knowledge. However, the role of a brief intermittent early exposure to milk in the neonatal unit is controversial[10,11] and might have a low effect, if any, on the development of atopy. Accordingly, we found it appropriate not to consider such intermittent exposures to CMP in this study".

Hypotheses

1. Does avoiding exposure to CMF for the first 3 days of life prevent development of FA later?

This is the main hypothesis of this study and was explained in the 'Background'.

Many Japanese maternity wards encourage BF, but allow mothers or nurses to supplement BF with CMF, e.g., approximately 6 to 10 hours after birth or even earlier, based on maternal preferences, but not based on clinical evidence. However, more than 20 to 30 years ago, sugar water was given instead of CMF supplement at birth. Thus, we hypothesized that early exposure to CMF at birth may be associated with the recent increase in children with FA. Therefore, a randomized clinical trial (RCT) was conducted to assess whether the risk of CMF sensitization and FA is decreased by either avoiding or supplementing CMF at birth.

3. Vitamin D hypotheses

Refer to the review article by Dr. G. Lack.[13]

The vitamin D hypothesis takes two forms: the vitamin D excess hypothesis argues that increases in vitamin D levels have led to increased allergies. The vitamin D deficiency hypothesis argues the opposite.

4. Dietary fat hypothesis

Refer to the review article by Dr. G. Lack.[13]

The dietary fat hypothesis argues that reduction in consumption of animal fats and a corresponding increase in the use of margarine and vegetable oils have led to the increase in allergies. The argument is that there has been an increase in the consumption of ω-6 polyunsaturated fatty acids, such as linoleic acid, and similarly that through reduced consumption of oily fish, there has been a reduction in ω-3

polyunsaturated fatty acids, such as eicosapentaenoic acid. ω-6 fatty acids lead to the production of prostaglandin E2 (PGE2), whereas ω-3 fatty acids inhibit synthesis of PGE2. PGE2 reduces interferon-γ production by T lymphocytes, thus resulting in increased IgE production by B lymphocytes. This has been proposed to explain the increase in the prevalence of asthma, eczema, and allergic rhinitis.

Objectives

Specific Aim 1: To compare atopic sensitization at 5-6 months and 2 years of age between BF with or without amino acid milk and BF with CMF.

Specific Aim 2: Subgroup analysis based on tertiles of serum levels of 25-hydroxyvitamin D (25OHD) at 5 months will be performed.

Exploratory Aim 1: To determine the relationships between serum levels of free fatty acids and FA and other atopic diseases.

Study design

Randomized, controlled, parallel two-group trial at a single institution

Pregnant women during the third trimester are randomly assigned to either BF with or without amino acid-based EF, called the BF±EF group, or BF plus CMF (≥5 ml/day from the first day of life to 1 month and ≥40 ml/day after 1 month until weaning), called the BF+CMF group, in a 1:1 ratio. Infants are followed until they are at least 2 years of age. Follow-up is extended 1 year if participating infants have FA. When the mother needs to add more than 150 ml/day EF for 3 consecutive days to BF because of a low amount of breast milk, EF is switched to CMF. Thus, the offspring allocated to BF±EF can avoid CMF for at least the first 3 days of life.

Subgroup

1. Tertiles of 25(OH)D levels at 5 months of age

Measure 25OHD levels in the serum obtained at 5 months of age.

Exploratory variables

1. Fatty acid levels in the serum, which are planned to report as another clinical study.

Measure fatty acid levels in the serum obtained at 5 months of age.

Twenty-four kinds of fatty acids such as arachidonic acid will be measured by high-performance liquid chromatography, which may be outsourced to SRL, using residual serum samples obtained at 5 months of age.

Research Implementation System

I. Clinical examination

a. Pediatrician: Dr. Mitsuyoshi Urashima: Enrollment and participants' clinical examination before allergic signs. If participating infants have any allergic signs, e,g., food-specific IgE ≥0.35 UA/ml blood sampling at 5 months of age, then they are referred to Dr. Tachimoto to diagnose atopic diseases and for follow-up.

   At the outpatient clinic of The Maternal and Child Center in Jikei University Hospital

b. Pediatrician: Dr. Hiroshi Tachimoto: Clinical examination after allergic signs and diagnosis of atopic diseases with or without the oral food challenge (OFC) test, management, and follow-up.

   At the outpatient clinic of Pediatrics in Jikei University Hospital

II. Endpoint committee

Among the following five members who do not know the allocation group and will not examine these participating infants at outpatient clinics, if ≥3 recognize the outcome as positive, the outcomes are finally determined to be positive.

1. Hidetoshi Mezawa, M.D., Ph.D.
2. Takashi Urashima, M.D., Ph. D.
3. Daishi Hirano, M.D., Ph.D.

4. Mai Okuyama, M.D.
5. Noriko Gocho, M.D.

Department of Pediatrics
The Jikei University School of Medicine
Tokyo, Japan

What the committee is expected to determine.

These five members determine whether the infant has had an episode of FA or not.

0. No FA
1. Immediate allergic reaction
2. Anaphylaxis

For 1 (and 2) above, they determine the status at the second birthday by reviewing detailed episodes, results of the OFC test, and levels of food-specific IgE.

1. Sustained unresponsiveness
   Cow's milk allergy, the infant can drink raw milk ≥50 ml
   Egg allergy, the infant can eat baked egg ≥30g,
   Wheat allergy, the infant can eat udon noodle ≥15g
2. Probable unresponsiveness, e.g., in the process of increasing dose of allergenic food, but not yet reach to predefined dose of unresponsiveness mentioned above.
3. Not yet unresponsive, when the infant eats, he or she still reacts to the allergenic food.
4. Not sure because the infant has not consumed the food allergen

The infants are still state of either 2, 3, or 4 above, they are considered not yet outgrow = remain food allergy.

III. Data monitoring

A) Randomization using a block of four by Dr. Akio Nakashima who does not interview participating families and does not examine their infants.

B) Data monitoring

If the frequency of adverse events deviates in either intervention group, the committee reports the data to the Safety and Data Monitoring Committee as well as the lead investigator.

a. Akio Nakashima, M.D., Ph.D. (physician and nephrologist)

b. Ms. Haruka Wada (secretary at Molecular Epidemiology)

Division of Molecular Epidemiology
The Jikei University School of Medicine
Tokyo, Japan

IV. Safety and Data Monitoring Committee

a. Judging from data of interim analysis, this committee may recommend stopping or continuing the trial.

b. Responding to a report of adverse events, the committee may recommend stopping the trial or allowing the trial to continue.

① Motohiro Ebisawa, M.D., Ph.D.
② Sakura Sato, M.D., Ph.D.
③ Kiyotake Ogura, M.D., Ph.D.
Department of Pediatrics
Sagamihara National Hospital
Kanagawa, Japan

Participants

Inclusion criteria

1. Be in a high-risk group for atopic sensitization: at least one of the father, mother, brothers, or sisters of infants has atopic diseases (asthma, atopic dermatitis, FA, allergic rhinitis, hay fever)

2. Obtained written informed consent.

a. Participants who understand randomization into two groups: BF with or without

amino acid milk and BF with CMF.

b. Blood examinations at 5-6 months and 2 years of age are required.

3. Be available to visit the outpatient clinic at 1 month, 2 months, 3 months, 4 months, 5 months, 7~8 months, 9~10 months, 12 months, 14~15 months, 18 months, and 24 months of age.

Exclusion criteria

1. Less than 36 weeks of gestational age at birth or birth weight less than 2000 g.
2. Having serious congenital anomalies.
3. Being a triplet or more: twins may participate.
4. Abnormal delivery such as asphyxia (Apgar score 6 or less at 5 minutes after birth)

Interventions

1. BF±EF

Mothers in this group are asked to maintain exclusive BF. However, if the amount of breast milk is not sufficient, then EF may be added until starting solid food. If the mother needs to supply EF more than 150 ml/day for 3 consecutive days in addition to BF because of a low amount of breast milk, EF is switched to CMF. Thus, the offspring allocated to BF±EF can avoid CMF for at least the first 3 days of life.

2. BF+CMF

Mothers in this group are asked to maintain exclusive BF, but are also asked to simultaneously supply a small amount of CMF ≥5 ml/day from the first day of life until 1 month, and ≥40 ml/day after 1 month until weaning. When infants are born after 18:00, CMF may be added on the 2nd day.

Both CMF (Hohoemi®) and EF (Meiji Elemental formula®) are purchased from Meiji Holdings Co. Ltd. (Chuo-ku, Tokyo, Japan).

EF (Meiji Elemental formula®)

For infants with FA, total proteins are replaced with refined amino acids. However, other fats, carbohydrates, vitamins, and minerals are basically the same as in CMF. Energy is

66 kcal/100 ml (17%) and 391 kcal/100 g powder.

Blinding to allocation group

a. Participating family: Not blinded

b. Dr. M.U.: Not blinded, because he needs to confirm compliance with the intervention by interviewing participating families at the outpatient clinic in the Maternal and Child Center of Jikei University Hospital. When an infant has food-specific IgE ≥0.10 U_A/ml at 5 months blood test, the infant is referred to Dr. H.T. at the outpatient clinic of Pediatrics without informing Dr. H.T. about the allocation group.

c. Dr. H.T.: Blinded, who is a specialist in pediatric allergic diseases, diagnoses FA, without knowing the allocation group.

d. Endpoint committee: Blinded and no contact with the participants.

Outcome Measures

Primary outcome

1. **Atopic sensitization:**

The primary outcome was atopic sensitization to cow’s milk protein, i.e., serum levels of cow’s milk-specific IgE (CM-IgE) ≥0·35 UA/ml at 24 months of age. Serum levels of CM-IgE at 5 and 24 months of age as a continuous variable were used as reference values for primary outcome. Other atopic sensitization to egg white and wheat ≥0·35 UA/ml at 24 months of age were also evaluated to be compared with cow’s milk sensitization. Outsourced for measuring with ImmunoCAP Specific IgE® (Thermo Fisher Diagnostics K.K., Minato-ku, Tokyo, Japan) to SRL Inc. and compared as both continuous and dichotomous (CM-IgE: ≥0.35 UA/ml) variable between BF±EF and BF+CMF group.

Secondary outcomes

1. **Immediate type of FA**

Presenting with at least one organ symptom of skin (urticaria, angioedema, erythema, pruritus, periorbital edema, etc.), respiratory (dry staccato cough,

hoarseness, wheezing, etc.), gastrointestinal (nausea, vomiting, colicky abdominal pain, etc.), or circulatory (tachycardia, hypotension, loss of consciousness, etc.) problems triggered by the food ingestion that appeared within a few hours, in combination with blood examination measuring serum levels of the suspected food-specific IgE: ≥0.35 U_A/ml by means of a fluorescence enzyme immunoassay.

In this trial, symptoms triggered by skin contact directly with food or indirectly with food-contaminated hands are not included.

The OFC test (Appendix 1) is performed around 1 year of age for infants who are sensitized to certain foods but for whom clinical reactivity after ingestion of the food allergen is unknown. Thus, immediate allergic reaction is a food-specific IgE: ≥0.35 U_A/ml plus either a FA episode or positive OFC test. FA is re-evaluated regarding whether the infant could eat the causative food allergen without an allergic reaction at the 2-year-old birthday.

2. Anaphylactic type of FA

When symptoms are derived from multiple (at least two of above) organs, e.g., urticaria and wheezing, the condition is defined as an anaphylactic reaction.

3. Desensitization of FA (= Sustained unresponsiveness or outgrow)

If infants, who had been once diagnosed as immediate type of FA especially with cow's milk allergy, egg allergy, and wheat allergy, can eat the allergenic food (raw milk ≥50 ml, baked egg 30g, udon noodle 15g) without allergic signs by 2nd birthday, then they are defined as 'sustained unresponsiveness = outgrow' to the food allergen in this trial.

4. Each food allergy

a. Cow's milk allergy was defined allergic reaction by OFC-test or cow's milk intake with or without heated or processed with cow's milk-specific IgE are positive (≥0.35 U_A/ml).

b. Egg allergy was defined allergic reaction by OFC-test or egg intake with or without heated or processed with egg white-specific IgE are positive (≥0.35 U_A/ml).

c. Wheat allergy was defined allergic reaction by OFC-test or wheat intake with or without heated or processed with wheat-specific IgE are positive (≥0.35 U_A/ml).

d. Other food allergy was defined allergic reaction by OFC-test or a certain food intake with or without heated or processed with the food-specific IgE are positive (≥0.35 U_A/ml).

Exploratory outcomes: which are planned to report in another opportunity.

1. Atopic dermatitis

Check the SCORAD index[14] at the 1- and 3-month check-ups.

2. Asthmatic bronchitis

A stringent index that includes frequent wheezing during the first 3 years of life and either one major risk factor (parental history of asthma or eczema) or two of three minor risk factors (eosinophilia, wheezing without colds, and allergic rhinitis).[15]

Major Criteria

1. Parental asthma†
2. Eczema diagnosed by a medical doctor‡
3. Sensitization with elevated mite- or house dust-specific IgE ≥0.10 U_A/ml

Minor Criteria

1. Allergic rhinitis diagnosed by a medical doctor§
2. Wheezing apart from colds
3. Eosinophilia (≥4%)

3. Sensitization to Japanese cedar pollen

Japanese cedar pollen-specific IgE ≥0.10 U_A/ml

4. Allergic rhinitis

Allergic rhinitis: Rhinitis signs and symptoms for 1 month and mite-specific IgE ≥0.10 U_A/ml

Safety outcomes

The following are obtained from an interview at the outpatient clinic just after the 2-year-old birthday.

1. Admission for any reason
2. Emergency room visit for any reason
3. Growth retardation: Height and weight at 2-year-old birthday

Flow of participants

1. Informed consent and registration

a. Pregnant women and their husbands are informed about the trial in detail at a childbirth class during late pregnancy through a talk by Dr. M. Urashima and the informed consent document. Written, informed consent is sent to the data monitoring center at the Molecular Epidemiology Division of Jikei University School of Medicine.

b. Current or previous family history of atopic diseases (asthma, atopic dermatitis, FA, allergic rhinitis, hay fever) is obtained through questionnaires as well as parental demographic information such as maternal age and body weight/height. Only pregnant women who are confirmed to have a family history of atopic diseases are included.

c. Food questionnaires of mothers (Appendix 3) are taken during the third trimester of pregnancy and at 1, 3, and 5 months of age after birth.

2. At the maternity ward of Jikei University Hospital

a. Some included pregnant women are excluded after delivery, due to, e.g., cleft palate.

b. Perinatal information, e.g., gestational weeks and birth weight, are taken from

charts.

c. Nursing staff is informed of the participants' names and to which group they are allocated.

d. Mothers are informed to which group they belong after delivery; thus, mothers are not blinded to the allocation group.

e. Mothers belonging to the BF+CMF group are asked to supplement BF with CMF at least 5 ml from the first day of delivery. However, if the delivery time is after 18:00, they are allowed to supply CMF in addition to BF starting at midnight of the 2nd day.

f. Mothers belonging to the BF±EF group may add amino acid-based EF when they feel that BF is not enough. If the mother adds more than 150 ml/day EF to BF for 3 consecutive days, EF is switched to CMF. Thus, offspring allocated to BF±EF can avoid CMF for at least the first 3 days of life.

g. Nurses at the maternity ward are allowed to feed babies instead of their mothers.

3. At the outpatient clinic in the Maternal and Child Center of Jikei University Hospital

a. Participating parent(s) are interviewed, and their children are examined at least seven times from 1, (2,) 3, (4,) 5-6, (7-8,) 9-10, 12, (14-15,) 18, and 24 months of age at the outpatient clinic of Jikei University Hospital by a study pediatrician (M.U.) who is not blinded to the allocation group.

b. If infants have exanthema, the pediatrician provides their parents clear guidance about aggressive skin care such as how to wash their face and body using soap and a shower by showing them a video.

c. Blood is sampled from all infants at 5-6 and 24 months.

4. At the outpatient clinic in Pediatrics of Jikei University Hospital

In case of at least one antigen-specific IgE is 0.1 IU_A/ml or more, the infant is referred to a study specialist of pediatric allergy (H.T.) at the same Jikei University Hospital who does not know the infant's allocation group.

5. Pediatrics Ward of Jikei University Hospital

The OFC test is performed at admission (Appendix 1) by pediatricians who are blinded

to the allocation group.

6. Efforts to reduce loss to follow-up

a. Using email, we try to contact participants who move to distant places regarding whether their infants have FA and make efforts to reduce the number of infants lost to follow-up, from whom IgE data are not obtained at 5 or 24 months.

b. If participants do not visit the outpatient clinic after their 2-year-old birthday and we receive no responses to the email, they are considered lost to follow-up.

7. Blood sampling

All infants undergo blood examinations to measure serum total and antigen-specific IgE.

a. At the 5- to 6-month check-up prior to first solid food introduction:
Egg white, cow’s milk, wheat, mites.

b. At 2 years of age:
Egg white, cow’s milk, wheat, mites, Japanese cedar pollen, dog dandruff, cat dandruff.

c. Unscheduled visits are made to the clinic when participating infants have atopic symptoms, particularly suspicious of FA.
e.g., exanthema just after eating an egg dish.

8. Randomization

a. EF and CMF

Both CMF (Hohoemi®) and EF (Meiji Elemental formula®) are purchased from Meiji Holdings Co. Ltd. (Chuo-ku, Tokyo, Japan).

b. Randomization

Pregnant women that meet the inclusion criteria are randomly assigned to either the BF+CMF or BF±EF group according to an identification number generated by computer based on permutated blocks of four that are determined beforehand (Appendix 2). Staff members at the maternity ward are informed of which intervention applies to each pregnant participant before hospitalization for childbirth.

c. Blinding

According to the nature of the intervention, we do not use double blinding. However, we try to minimize bias using the following strategies:

Primary outcome: Cow’s milk-specific IgE as an objective indicator.

Secondary outcomes: determine by prospective, randomized, open, blinded-endpoint (PROBE), determined by the endpoint committee members who do not examine the infants or are blinded to the allocation group’.

9. Compliance with CMF or EF

1. At the maternity ward, every day’s CMF or EF intake is monitored by each participant (mother). The nurse may help with the intervention and monitoring.
2. At the outpatient clinic, mothers are asked whether they are using CMF, EF, or exclusive BF.

10. Monitoring of secondary outcomes

a. Scheduled

At either the Maternal and Child Center or Pediatrics of Jikei University Hospital, participating infants are examined frequently.

b. Unscheduled

If the infants have any allergic signs, e.g., urticaria after eating allergenic food, they are advised to call Jikei University Hospital. H.T. or other pediatricians try to examine the infants on the day or next day and perform a blood examination.

Statistical analysis

1. Sample size

300

2. Sample size calculation

300 pregnant women and their offspring

We hypothesized that cow's milk sensitization at 2 years of age, i.e., cow's milk-specific IgE ≥0.35 U_A/ml, would be present in 10% of one group of infants and 25% of the other group, with a type I error (two-sided) of 5% and a power of 90%, on the assumption of 3% loss to follow-up. Therefore, 1:1 divided samples of approximately 300 participants were calculated as sufficient to detect a significant difference.

```
. sampsi .1 .25

Estimated sample size for two-sample comparison of proportions

Test Ho: p1 = p2, where p1 is the proportion in population 1
                        and p2 is the proportion in population 2
Assumptions:

         alpha =   0.0500  (two-sided)
         power =   0.9000
            p1 =   0.1000
            p2 =   0.2500
         n2/n1 =   1.00

Estimated required sample sizes:

            n1 =      146
            n2 =      146
```

n = 292

Assuming 3% loss to follow-up, 300 are needed to enroll.

3. Study period

We assume that 100 pregnant women per year will participate in this trial. Therefore, the accrual period will be 3 years. The last infant is followed up 2 years later. Thus, a total of 5 years is predicted to be needed to accomplish this trial.

4. Interim analysis

Interim analyses are planned two times at reaching 100 and 200 participating infants who had their second birthday. The P value for significance at the interim analysis is set as <0.001 according to the Peto's stopping boundaries.

5. Planned methods of analysis

Outcomes are analyzed by intention-to-treat analyses between the two comparative groups.

a. Serum levels of cow's milk-specific IgE at 5 months and 24 months of age as a continuous variable are compared using Mann-Whitney test.
b. Risk of cow's milk-specific IgE ≥0.35 U_A/ml will be analyzed with a 2x2 table with the chi-square test and represented by the risk ratio (RR), and 95% confidence interval (95% CI).
c. Secondary outcomes will be compared with RR, and 95% CI.
d. Safety outcomes will be compared with RR, RD and 95% CI or Student's t test.
e. Subgroup analysis will be done according to tertiles of 25(OH)D.
f. All reported P values will be two-sided.
g. Values with $P < 0.05$ will be considered statistically significant.
h. All data will be statistically analyzed using Stata 14.0 (StataCorp LP., College Station, TX, USA).

6. Subgroup analyses

To clarify whether BF±EF or BF+CMF significantly affects the subgroups of 25(OH)D (e.g., tertile, quartile, quintile), P for interaction ($P_{interaction}$) is computed by creating multiplicative variables. The results of these analyses are not corrected for multiple comparisons.

Safety

Many Japanese maternity wards recommend BF, but allow mothers or nurses to help by supplying CMF to supplement BF after feeding colostrum, e.g., 6 to 10 hours after birth.

This is a food intervention trial. Thus, basically no safety concerns exist. However, the following are compared.

a. Admission due to any disease
b. Emergency visit or admission for any reason
c. Body height and weight at 24 months of age

Early withdrawal

Unknown serious adverse events.

Compensation

Mitsui Sumitomo Insurance Fire provides liability insurance (NB41027726) for the principal investigator, to compensate for costs of treating disability as a result of side effects caused by consuming milk.

The maximum amount of compensation is 100,000,000 yen per person and 300,000,000 yen per trial.

Consideration concerning the protection of human rights and privacy

1. Written informed consent is obtained after sufficient explanation.
2. Participants can withdraw from the trial after providing written, informed consent.
3. Withdrawal is not considered a disadvantage for participants.
4. Private information is exchanged with study ID at the Jikei University School of Medicine; therefore, private information cannot leak from there (linkable anonymizing).

Publication of research results

We aim to publish the results of this trial in a scientific journal or present them at academic conferences. Personal information will be carefully concealed when we present the results.

Research funds

1. JSPS KAKENHI Grant Number JP16K09074

2. the Practical Research Project for Allergic Disease and Immunology of the Japan Agency for Medical Research and Development, AMED (15ek0410019h0101)
3. Dairy Products Health Science Council and Japan Dairy Association

Attribution of intellectual property rights

If intellectual property rights such as patent rights become relevant, such rights will be attributable to the investigator.

References

1. Gupta R, Sheikh A, Strachan DP, Anderson HR. Time trends in allergic disorders in the UK. Thorax. 2007 Jan;62(1):91-6.
2. Lin RY, Anderson AS, Shah SN, Nurruzzaman F. Increasing anaphylaxis hospitalizations in the first 2 decades of life: New York State, 1990 -2006. Ann Allergy Asthma Immunol. 2008 Oct;101(4):387-93.
3. Poulos LM, Waters AM, Correll PK, Loblay RH, Marks GB. Trends in hospitalizations for anaphylaxis, angioedema, and urticaria in Australia, 1993-1994 to 2004-2005. J Allergy Clin Immunol. 2007 Oct;120(4):878-84.
4. American Academy of Pediatrics. Committee on Nutrition. Hypoallergenic infant formulas. Pediatrics. 2000 Aug;106(2 Pt 1):346-9.
5. Greer FR, Sicherer SH, Burks AW; American Academy of Pediatrics Committee on Nutrition; American Academy of Pediatrics Section on Allergy and Immunology. Effects of early nutritional interventions on the development of atopic disease in infants and children: the role of maternal dietary restriction, breastfeeding, timing of introduction of complementary foods, and hydrolyzed formulas. Pediatrics. 2008 Jan;121(1):183-91.
6. Global strategy for infant and young child feeding. Geneva: World Health Organization,2003(http://apps.who.int/iris/bitstream/10665/42590/1/9241562218.pdf?ua=1&ua=1).
7. Katz Y, Rajuan N, Goldberg MR, Eisenberg E, Heyman E, Cohen A, Leshno M. Early exposure to cow's milk protein is protective against IgE-mediated cow's milk protein allergy. J Allergy Clin Immunol. 2010 Jul;126(1):77-82.
8. Koletzko S, Filipiak-Pittroff B, von Berg A, Grübl A, Heinrich J, Krämer U, Wichmann HE, Bauer CP, Reinhardt D, Berdel D; German Infant Nutritional Intervention Study Group. Supplementation with cow's milk at birth is not recommended. J Allergy Clin Immunol. 2011 Mar;127(3):836-8
9. Lindfors A, Enocksson E. Development of atopic disease after early administration of cow milk formula. Allergy. 1988 Jan;43(1):11-6.
10. de Jong MH, Scharp-van der Linden VT, Aalberse RC, Oosting J, Tijssen JG, de Groot CJ. Randomised controlled trial of brief neonatal exposure to cows' milk on the

development of atopy. Arch Dis Child. 1998 Aug;79(2):126-30.

11. Saarinen KM, Juntunen-Backman K, Järvenpää AL, Kuitunen P, Lope L, Renlund M, Siivola M, Savilahti E. Supplementary feeding in maternity hospitals and the risk of cow's milk allergy: A prospective study of 6209 infants. J Allergy Clin Immunol. 1999 Aug;104(2 Pt 1):457-61.
12. Høst A, Husby S, Osterballe O. A prospective study of cow's milk allergy in exclusively breast-fed infants. Incidence, pathogenetic role of early inadvertent exposure to cow's milk formula, and characterization of bovine milk protein in human milk. Acta Paediatr Scand. 1988 Sep;77(5):663-70.
13. Lack G. Epidemiologic risks for food allergy. J Allergy Clin Immunol. 2008 Jun;121(6):1331-6.
14. No author listed. Severity scoring of atopic dermatitis: the SCORAD index. Consensus Report of the European Task Force on Atopic Dermatitis. Dermatology. 1993;186(1):23-31.
15. Castro-Rodríguez JA, Holberg CJ, Wright AL, Martinez FD. A clinical index to define risk of asthma in young children with recurrent wheezing. Am J Respir Crit Care Med. 2000 Oct;162(4 Pt 1):1403-6.

Appendix 1

Table 1. Oral Food Challenge (OFC) test

Oral Food Challenge (OFC) test				
Hen's egg	STEP①	egg yolk x 1 (As pumpkin cake)	70	g
	STEP②	whole egg x 1/2 (As pumpkin cake)	70	g
	STEP③	whole egg x 1 (As scrambled egg)	45	g
ilk and its produc	STEP①	Heated milk (As pumpkin cake)	1	ml
	STEP②	Heated milk (As pumpkin cake)	10	ml
	STEP③	Heated milk (As potage soup)	50	ml
	STEP④	Yogurt	48	ml
Wheat	STEP①	Flour (As pumpkin cake)	1	g
	STEP②	Flour (As pumpkin cake)	5	g
	STEP③	Flour (As pumpkin cake)	15	g
	STEP④	Flour (As pumpkin cake)	30	g
	Udon dried noodles 3g = Flour 1g = wheat protein 0.08g			
	Udon dried noodles 15g = Flour 5g = wheat protein 0.4g			
	Udon dried noodles 50g = Flour 15g = wheat protein1.3g			
	Udon dried noodles 100g = Flour 30g = wheat protein 2.(			
Soy bean	STEP①	Tofu	100	g
peanuts	STEP①	Peanuts	2	g
	STEP②	Peanuts	10	g
almond	STEP①	Almond	2	g
	STEP②	Almond	10	g
walnuts	STEP①	Walnuts	2	g
	STEP②	Walnuts	10	g
Cashew Nuts	STEP①	Cashew Nuts	2	g
	STEP②	Cashew Nuts	10	g
Sesame (other kinds of nuts)	STEP①	Sesame	2	g
	STEP②	Sesame	10	g
Soba	STEP①	Soba	15	g
	STEP②	Soba	50	g
	STEP③	Soba	100	g
Meat		Boiled meat	50	g
Fish		Boiled Fish	40	g
ustacean Soft bo		Shrimp, Crab, Squid, Octopus (served as Hamburger)	15	g
Shellfish		Clams, Scallop, etc. (served as hamburger)	20	g
fish egg		Baked Tarraco	20	g
		Salmon roe	10	g
Potatoes		Boiled Potato Soup	100	g
corn		Corn compote	30	g
fruits		Strawberry Puree	120	ml

Appendix 2

Table 2. Permutated blocks of four

1	2	3	4	5	6
A	A	P	P	A	P
A	P	A	P	P	A
P	P	A	A	A	P
P	A	P	A	P	A

Appendix 3

Questionnaires about food intake

If you obtained written informed consent from the participant, please send the following information by FAX.

Date:_______year,_______month,_______day

Person who filled out the form:

✎ During the last month, how often did you eat each kind of food per week? Please check (☑).

	Rarely	Once per week	Several times per week	Once per day	Twice per day	Three times per day
Example	☐	☐	☑	☐	☐	☐
Place to eat meal						
Restaurant (eating out)	☐	☐	☐	☐	☐	☐
Ramen shop (eating out)	☐	☐	☐	☐	☐	☐
Fast food (hamburger)	☐	☐	☐	☐	☐	☐
Fast food (rice bowl)	☐	☐	☐	☐	☐	☐
Fast food (soba, udon)	☐	☐	☐	☐	☐	☐
Lunch box bought at convenience store	☐	☐	☐	☐	☐	☐
Cafeteria at work place	☐	☐	☐	☐	☐	☐
At home	☐	☐	☐	☐	☐	☐
Foods						

Butter	▢	▢	▢	▢	▢	▢
Margarine	▢	▢	▢	▢	▢	▢
Vegetable oil	▢	▢	▢	▢	▢	▢
Vegetable oil<br>Please check the most appropriate answer	Safflower oil	Canola oil	Sesame oil<br>Soy oil<br>Sunflower oil	Peanut oil, Coconut oil, Corn oil, Palm oil	others	
Olive oil	▢	▢	▢	▢	▢	▢
Biscuits[*1]	▢	▢	▢	▢	▢	▢
Junk food (potato chips, etc.)	▢	▢	▢	▢	▢	▢
Chocolate	▢	▢	▢	▢	▢	▢
Mayonnaise[*2]	▢	▢	▢	▢	▢	▢
Plain bread	▢	▢	▢	▢	▢	▢
Pastry	▢	▢	▢	▢	▢	▢
Croissant	▢	▢	▢	▢	▢	▢
Other kinds of bread						
Instant noodles	▢	▢	▢	▢	▢	▢
Fried food at home (tempura,fried chicken, fried potatoes, etc.)	▢	▢	▢	▢	▢	▢
Fried food outside (tempura, fried chicken, fried potatoes, etc.)	▢	▢	▢	▢	▢	▢
Beef (including hamburger)[*3]	▢	▢	▢	▢	▢	▢
Processed meat (sausage, salami, hot dogs, bacon, etc.)	▢	▢	▢	▢	▢	▢

Cow's milk	▢	▢	▢	▢	▢	▢
Cheese	▢	▢	▢	▢	▢	▢
Creams (include cream for coffee)	▢	▢	▢	▢	▢	▢
Ice cream	▢	▢	▢	▢	▢	▢
Pork	▢	▢	▢	▢	▢	▢
Chicken	▢	▢	▢	▢	▢	▢
Heated eggs	▢	▢	▢	▢	▢	▢
Raw eggs	▢	▢	▢	▢	▢	▢
Fish eggs (salmon roe, flying fish roe, roe, sea urchin, dried mullet roe, caviar, etc.)	▢	▢	▢	▢	▢	▢
Soy (including soy food, natto, tofu, etc.)	▢	▢	▢	▢	▢	▢
Soba	▢	▢	▢	▢	▢	▢
Tuna (canned tuna, sashimi, etc.)	▢	▢	▢	▢	▢	▢
Swordfish	▢	▢	▢	▢	▢	▢
Salmon (including canned salmon)	▢	▢	▢	▢	▢	▢
Mackerel, sardine, horse mackerel, Pacific saury (including canned)	▢	▢	▢	▢	▢	▢
White fish (red snapper, etc.)	▢	▢	▢	▢	▢	▢
Peanuts (including peanut butter)	▢	▢	▢	▢	▢	▢
Walnuts	▢	▢	▢	▢	▢	▢
Almonds	▢	▢	▢	▢	▢	▢
Cashew nuts	▢	▢	▢	▢	▢	▢
Macadamia nuts	▢	▢	▢	▢	▢	▢

Hazelnuts	▢	▢	▢	▢	▢	▢
Coconut (including processed food)	▢	▢	▢	▢	▢	▢
Other kinds of nuts						
Supplement						

*1. Biscuits include cream puffs, sponge cake, donuts, etc.

*2. Mayonnaise includes creamy salad dressing.

*3. Beef includes heart, liver, diaphragm, and stomach.

Summary of changes to
the Trial Protocol and Statistical Analysis Plan (SAP)

Ver. 1. 2013.8.1

Ver. 2. 2016.7.22

Ver.3. 2018.4.1

Ver. 2: certified on 2016.7.22 by the Jikei ethics committee.

1. Measurement of serum levels of free fatty acids

As exploratory analyses, we decided to analyze serum levels of free fatty acids using the residual serum samples stocked in deep freezers (−80°C).

Associations among food intake questionnaires, serum levels of free fatty acids, and FA are analyzed, as exploratory study.

We added newly followings in SAP.

a. Associations between free fatty acids and FA are analyzed.

b. Associations between answers to questionnaires on food intake and FA risks are explored.

c. Associations between answers to questionnaires on food intake and free fatty acids are explored.

d. Associations between 25(OH)D levels and growth at the 2-year-old birthday will be compared.

2. Key secondary outcomes were rearranged.

Definition was not changed but separated into secondary outcomes and exploratory outcomes. In addition, each food allergy was made clear.

From;

Key secondary outcomes

1. immediate type of food allergy including anaphylaxis
2. worsening of atopic dermatitis
3. desensitization of food allergy
4. atopic sensitization alone
5. asthmatic bronchitis

To;

Secondary outcomes: food allergy related

I. Immediate type of food allergy (FA)
II. Anaphylactic type of FA
II. Desensitization of FA (= Sustained unresponsiveness or outgrow)
III. Atopic sensitization alone
III. Each food allergy
    a. Cow’s milk allergy
    b. Egg allergy
    c. Wheat allergy
    d. Other food allergy

Exploratory outcomes: atopic conditions other than food allergy

I. Atopic dermatitis
II. Asthmatic bronchitis
III. Sensitization to Japanese cedar pollen
IV. Allergic rhinitis

Ver. 3: certified on 2018.4.1, to increase the credibility of the secondary outcomes.

1. New members in the endpoint committee

We added one general pediatrician and one specialist in pediatric allergies to the endpoint committee.

From;

a. Hidetoshi Mezawa, M.D., Ph.D.,
b. Takashi Urashima, M.D., Ph.D.,
c. Daishi Hirano, M.D., Ph.D

To;

a. Hidetoshi Mezawa, M.D., Ph.D.,
b. Takashi Urashima, M.D
c. Daishi Hirano, M.D., Ph.D
d. Mai Okuyama, M.D
e. Noriko Gocho, M.D.
