# Supplementary material for: Effect of Avoiding Cow's Milk Formula at Birth on Prevention of Asthma or Recurrent Wheeze Among Young Children: Extended Follow-up From the ABC Randomized Clinical Trial
Source: JAMA Netw Open. 2020 Oct 2;3(10):e2018534. doi: 10.1001/jamanetworkopen.2020.18534 (PMC7532386; doi:10.1001/jamanetworkopen.2020.18534)
Supplement: Supplement 2. — eFigure 1. Histograms of 25(OH)D levels at 5 months and 24 months of age eFigure 2. Comparison of 25(OH)D levels at 5 and 24 months of age among 4 groups eTable 1. Interaction between tertiles of 25(OH)D levels and avoiding CMF at birth eTable 2. Interactions between each allergen-specific IgE level at 5 months of age and the intervention eTable 3. Incidence of asthma or recurrent wheeze by duration of adherence to the BF/EF intervention [file jamanetwopen-e2018534-s002.pdf]

Supplementary Online Content

Tachimoto H, Imanari E, Mezawa H, et al. Effect of avoiding cow's milk formula at birth on prevention of asthma or recurrent wheeze among young children: extended follow-up from the ABC randomized clinical trial. *JAMA Netw Open*. 2020;3(10):e2018534. doi:10.1001/jamanetworkopen.2020.18534

eFigure 1. Histograms of 25(OH)D levels at 5 months and 24 months of age

eFigure 2. Comparison of 25(OH)D levels at 5 and 24 months of age among 4 groups

eTable 1. Interaction between tertiles of 25(OH)D levels and avoiding CMF at birth

eTable 2. Interactions between each allergen-specific IgE level at 5 months of age and the intervention

eTable 3. Incidence of asthma or recurrent wheeze by duration of adherence to the BF/EF intervention

This supplementary material has been provided by the authors to give readers additional information about their work.

© 2020 Tachimoto H et al. *JAMA Network Open*.

eFigure 1. Histograms of 25(OH)D levels at 5 months and 24 months of age

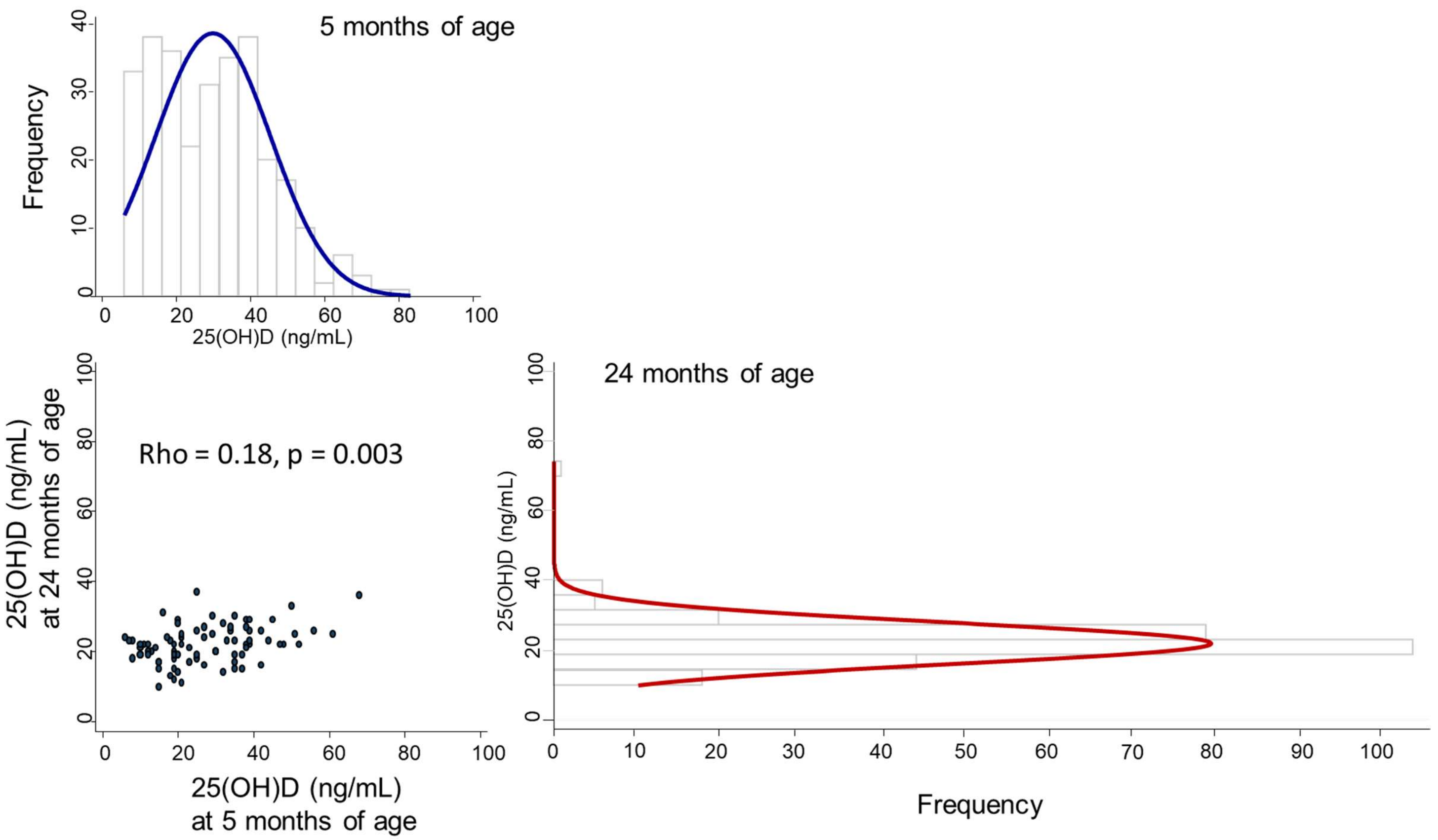

Spearman's rank correlation coefficient (rho) with linear regression was used to quantify the strengths of associations between two continuous variables: rho ≥0.4, strong; 0.4> rho ≥0.2, moderate; and rho <0.2, weak.

© 2020 Tachimoto H et al. *JAMA Network Open*.

eFigure 2. Comparison of 25(OH)D levels at 5 and 24 months of age among 4 groups

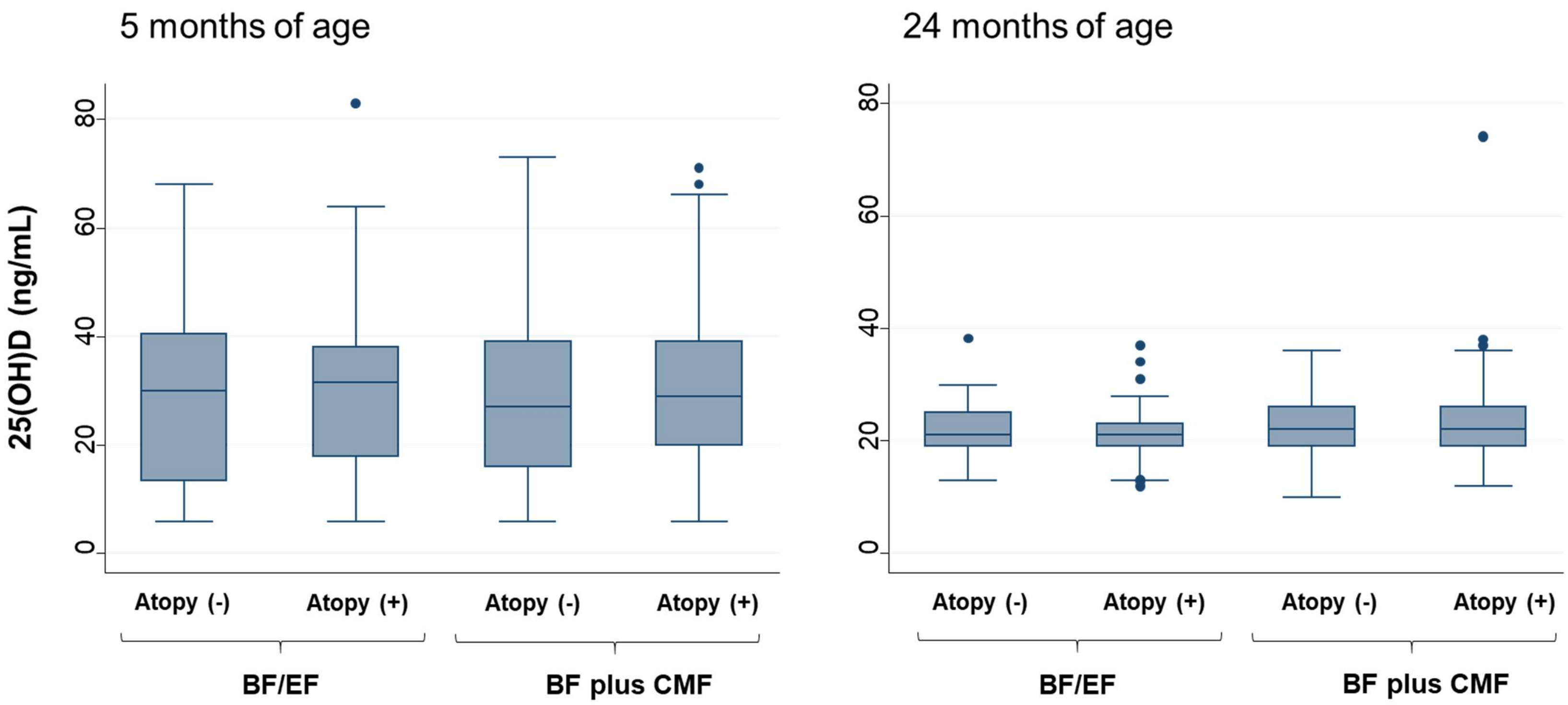

1) BF/EF without atopic conditions; 2) BF/EF with atopic conditions; 3) BF plus CMF without atopic conditions; 4) BF plus CMF with atopic conditions. The differences were assessed by the Kruskal-Wallis test.

© 2020 Tachimoto H et al. *JAMA Network Open.*

eTable 1. Interaction between tertiles of 25(OH)D levels and avoiding CMF at birth

	BF/EF	BF plus CMF	RD (95% CI)	RR (95% CI)	P for interaction
25(OH)D level at 5 months of age					.65
Tertile 1, 6-20 ng/mL, n = 99	5 (10.4)	7 (13.7)	-0.03 (-0.16 to 0.09)	0.76 (0.26-2.23)	
Tertile 2, 21-36 ng/mL, n = 96	3 (6.1)	8 (17.0)	-0.11 (-0.24 to 0.02)	0.36 (0.10-1.27)	
Tertile 3, 37-83 ng/mL, n = 98	5 (10.2)	11 (22.4)	-0.12 (-0.27 to 0.02)	0.45 (0.17-1.21)	
25(OH)D levels at 24 months of age					.25
Tertile 1, 10-19 ng/mL, n = 89	8 (16.3)	9 (22.5)	-0.06 (-0.23 to 0.10)	0.73 (0.31-1.71)	
Tertile 2, 20-23 ng/mL, n = 103	2 (3.9)	10 (19.2)	**-0.15 (-0.27 to -0.03)**	**0.20 (0.05-0.89)**	
Tertile 3, 24-74 ng/mL, n = 86	5 (12.8)	7 (15.2)	-0.02 (-0.17 to 0.12)	0.84 (0.29-2.44)	

When both sides of the 95% CI of the RD are more or less than 0 or both sides of the 95% CI of the RR do not include 1, the risk estimate is considered to be significant and is indicated as bold.

© 2020 Tachimoto H et al. *JAMA Network Open*.

eTable 2. Interactions between each allergen-specific IgE level at 24 months of age and the intervention

	BF/EF	BF plus CMF	RD (95% CI)	RR (95% CI)	P for interaction
Mite-specific IgE					.03
Low (<0.1 U_A/mL), n = 225	13 (11.5)	15 (12.8)	-0.02 (-0.11 to -0.07)	0.86 (0.43 to 1.72)	
High (≥0.1 U_A/mL), n = 59	2 (6.7)	12 (41.4)	**-0.35 (-0.55 to -0.15)**	**0.16 (0.04 to 0.66)**	
Milk-specific IgE					.03
Low (<0.1 U_A/mL), n = 168	11 (11.8)	7 (9.3)	0.02 (-0.07 to 0.12)	1.27 (0.52 to 3.11)	
High (≥0.1 U_A/mL), n = 117	4 (8.2)	20 (29.4)	**-0.21 (-0.35 to -0.08)**	**0.28 (0.10 to 0.76)**	
Egg white-specific IgE					.68
Low (<0.1 U_A/mL), n = 125	3 (4.8)	7 (11.1)	-0.21 (-0.35 to -0.08)	0.44 (0.12 to 1.61)	
High (≥0.1 U_A/mL), n = 161	12 (14.8)	20 (25.0)	-0.10 (-0.22 to 0.02)	0.59 (0.31 to 1.13)	
Wheat-specific IgE					.04
Low (<0.1 U_A/mL), n = 219	15 (10.1)	23 (16.1)	-0.01 (-0.09 to 0.08)	0.94 (0.44 to 2.00)	
High (≥0.1 U_A/mL), n = 56	0 (0.0)	4 (57.1)	**-0.36 (-0.56 to -0.15)**	**0.18 (0.04 to 0.71)**	

When both sides of the 95% CI of the RD are more or less than 0 or both sides of the 95% CI of the RR do not include 1, the risk estimate is considered to be significant and is indicated as bold.

© 2020 Tachimoto H et al. *JAMA Network Open*.

eTable 3. Incidence of asthma or recurrent wheeze by duration of adherence to the BF/EF intervention

	BF/EF n = 151		
Day of starting the addition of CMF to BF after birth<br>Total, n	Remained on BF/EF<br>n = 40	Day 15 or after<br>n = 41	Days 2[*1] - 14<br>n = 70
Asthma and recurrent wheeze, n (%)	5 (12.5)	3 (7.3)	7 (10.0)
95% confidence interval, %	4.2 to 26.8	1.5 to 19.9	4.1 to 19.5

*1. Three mothers in the BF/EF group mistakenly gave CMF to their infants from the second day after birth instead of the 4th day as the earliest start.

© 2020 Tachimoto H et al. *JAMA Network Open*.
