# Supplementary material for: Effect of Avoiding Cow's Milk Formula at Birth on Prevention of Asthma or Recurrent Wheeze Among Young Children: Extended Follow-up From the ABC Randomized Clinical Trial
Source: JAMA Netw Open. 2020 Oct 2;3(10):e2018534. doi: 10.1001/jamanetworkopen.2020.18534 (PMC7532386; doi:10.1001/jamanetworkopen.2020.18534)
Supplement: Supplement 3. — Data Sharing Statement [file jamanetwopen-e2018534-s003.pdf]

Data Sharing Statement

Data
Data available: Yes
Data types: Deidentified participant data
How to access data: urashima@jikei.ac.jp
When available: With publication

Supporting Documents
Document types: None

Additional Information
Who can access the data: Mitsuyoshi Urashima
Types of analyses: individual participants data meta analysis
Mechanisms of data availability: after approval of a proposal
